# Biomimetic Electronic Skin through Hierarchical Polymer Structural Design

**DOI:** 10.1002/advs.202309006

**Published:** 2023-12-10

**Authors:** Mengnan Zhang, Shu Gong, Karen Hakobyan, Ziyan Gao, Zeyu Shao, Shuhua Peng, Shuying Wu, Xiaojing Hao, Zhen Jiang, Edgar H. Wong, Kang Liang, Chun H. Wang, Wenlong Cheng, Jiangtao Xu

**Affiliations:** ^1^ Centre for Advanced Macromolecular Design and Australian Centre for NanoMedicine, School of Chemical Engineering UNSW Sydney NSW 2052 Australia; ^2^ Department of Chemical & Biological Engineering Monash University Clayton VIC 3800 Australia; ^3^ School of Mechanical and Manufacturing Engineering UNSW Sydney NSW 2052 Australia; ^4^ School of Engineering Macquarie University Sydney NSW 2109 Australia; ^5^ Australian Centre for Advanced Photovoltaics, School of Photovoltaic and Renewable Energy Engineering UNSW Sydney NSW 2052 Australia; ^6^ School of Mechanical, Materials and Mechatronic Engineering University of Wollongong Wollongong NSW 2522 Australia; ^7^ School of Chemical Engineering and Graduate School of Biomedical Engineering UNSW Sydney NSW 2052 Australia

**Keywords:** artificial skin, electronic skin, hydrogel, interfacial precipitation polymerization, polymer nanoparticles, triboelectric nanogenerator, water loss prevention

## Abstract

Human skin comprises multiple hierarchical layers that perform various functions such as protection, sensing, and structural support. Developing electronic skin (E‐skin) with similar properties has broad implications in health monitoring, prosthetics, and soft robotics. While previous efforts have predominantly concentrated on sensory capabilities, this study introduces a hierarchical polymer system that not only structurally resembles the epidermis‐dermis bilayer structure of skin but also encompasses sensing functions. The system comprises a polymeric hydrogel, representing the “dermis”, and a superimposed nanoporous polymer film, forming the “epidermis”. Within the film, interconnected nanoparticles mimic the arrangement of interlocked corneocytes within the epidermis. The fabrication process employs a robust in situ interfacial precipitation polymerization of specific water‐soluble monomers that become insoluble during polymerization. This process yields a hybrid layer establishing a durable interface between the film and hydrogel. Beyond the structural mimicry, this hierarchical structure offers functionalities resembling human skin, which includes (1) water loss protection of hydrogel by tailoring the hydrophobicity of the upper polymer film; (2) tactile sensing capability via self‐powered triboelectric nanogenerators; (3) built‐in gold nanowire‐based resistive sensor toward temperature and pressure sensing. This hierarchical polymeric approach represents a potent strategy to replicate both the structure and functions of human skin in synthetic designs.

## Graphical abstract

A hierarchical polymer system with a bilayer structure is successfully fabricated through a simple and robust in situ interfacial precipitation polymerization process. This bilayer structure closely mimics the structure of the epidermis and dermis in human skin. Integrating a gold nanowire‐based temperature and pressure sensor yield a biomimetic electronic skin with built‐in sensory functions resembling human skin.

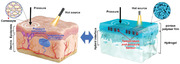

## 1 Introduction

**Figure 1:**
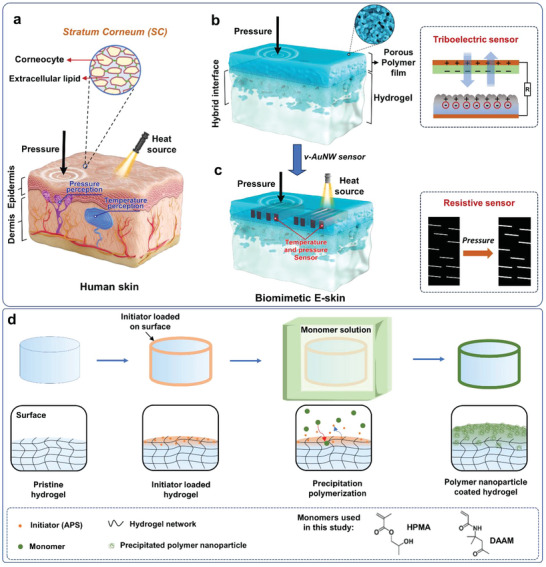
Human skin with various sensory receptors as inspiration for the design of biomimetic electronic skin (E‐skin) prepared through an in situ interfacial precipitation polymerization. a) Structure of human skin indicating the two key layers (epidermis and dermis), receptors, and the microstructure of the *stratum corneum* (SC, the outermost layer of the epidermis). b) Structure of a polymer nanoparticle coated hydrogel that possesses a comparable bilayer structure to the epidermis and dermis, porous polymer film layer (vs the epidermis), its interconnected nanoparticular structure (vs the interlocked corneocyte structure in the SC layer of the epidermis), and the hydrogel layer (vs the dermis). This polymer system can be directly employed as an E‐skin pressure sensor through a triboelectrification mechanism. c) Structure of biomimetic E‐skin prepared by integrating vertically aligned gold nanowire (v‐AuNW) sensors into a polymer nanoparticle‐coated hydrogel to sense temperature and pressure. This E‐skin works through a mechanism of resistance change upon pressure or temperature. d) Two‐step soaking‐polymerization procedure for producing polymer nanoparticle coated hydrogels: soaking the gel with a radical initiator (ammonium persulfate, APS) and then precipitation polymerization. For clarity, components in the polymerization such as the co‐initiator (*N,N,N’,N’*‐tetramethylethylenediamine, TMEDA) and crosslinker (*N,N’*‐methylenebisacrylamide, MBAA) are excluded from the figure.

Skin is the largest organ of the human body consisting of a hierarchical structure and sensory system. The top two layers of human skin are epidermis and dermis as shown in **Figure** **1**a. The epidermis, ranging in thickness from 20 to 500 µm, acts as a biophysical barrier against the external environment.^[^ ^1^, ^2^ ^]^ The epidermal barrier resides in the most superficial layer of the skin, the *stratum corneum* (SC). The SC exhibits a microstructure consisting of interlocked disc‐like corneocytes embedded in an extracellular lipid matrix (Figure 1a).^[^ ^3^, ^4^, ^5^ ^]^ The underlying layer of the epidermis is the dermis, which holds most of the skin's water and connects to the epidermis via a continuous epidermal‐dermal junction. The dermis consists of connective tissue that serves as a cushion from stress and strain.^[^ ^2^, ^6^ ^]^ This epidermis‐dermis bilayer structure contains various sensory receptors that allow the body to perceive external stimuli, such as pressure and temperature.

To mimic the unique structure of human skin, two layers of materials with distinct properties are typically employed. Hydrogels are commonly used to emulate the dermis because of their 3D tissue‐like characteristics, including intrinsically high water content, flexibility, and tunable elastic modulus.^[^ ^6^, ^7^, ^8^, ^9^, ^10^, ^11^, ^12^, ^13^, ^14^, ^15^, ^16^, ^17^ ^]^ Various materials have been used to fabricate mimics of the epidermis, including inorganic materials^[^ ^18^, ^19^, ^20^ ^]^ and elastomers^[^ ^21^, ^22^, ^23^, ^24^, ^25^ ^]^ (e.g., polydimethylsiloxane, PDMS). Inorganic materials such as carbon,^[^ ^19^, ^26^ ^]^ silica,^[^ ^27^ ^]^ and metallic materials^[^ ^28^, ^29^ ^]^ are able to reproduce the nanostructure of the epidermis and provide protective and self‐healing properties as well as electric conductivity that is responsive toward deformation or other external stimuli for fabricating sensory systems. Alternatively, integrating these materials into flexible substrates (e.g., hydrogels) can yield multilayer inorganic–organic composites to serve as artificial skin electronics. Elastomers have been commonly employed as a base when fabricating such soft electronics due to their chemical stability and comparable elastic modulus to skin. A bilayer hydrogel–elastomer hybrid that mimics the organization of the native epidermis and dermis has been widely recognized to integrate the properties of both hydrogels and elastomers.^[^ ^21^, ^30^, ^31^, ^32^ ^]^

Despite successes toward mimicking the epidermis‐dermis structure, bonding elastomers (or inorganic materials) onto hydrogels remains challenging due to their low surface energy and general chemical inertness. This often translates to specific and/or harsh conditions required for bonding, such as chemical crosslinking^[^ ^21^, ^33^, ^34^ ^]^ or adhesives^[^ ^35^, ^36^ ^]^ Therefore, the development of simple and versatile methodologies for hydrogel‐based skin mimics, especially for the epidermis, is highly desirable. Additionally, while hydrogel–elastomer hybrids exhibit sufficient softness and resilience to stresses, the elastomers in the hybrids generally possess low permeability towards body fluids and nutrients due to their hydrophobicity.^[^ ^6^ ^]^ Native epidermis typically achieves this through a nanoparticular morphology. Therefore, a breathable coating on hydrogel with nano‐pores resembling the epidermis, as opposed to a pure elastomer, is essential to incorporate permeability that allows for the diffusion of small molecules (e.g., water).^[^ ^6^ ^]^ It, however, remains a challenge to fabricate durable films on soft hydrogel materials. This is well‐known to be due to their low surface energy and low mass content. Moreover, the human skin contains various sensory receptors that allow the body to sense pressure, pain, temperature, etc. Mimicking these features generally requires the integration of components such as sensors, a power supply, and wireless signal transducers into hydrogels. To the best of our knowledge, building a biomimetic hydrogel system with such structural features and integrated sensing capability has not been achieved yet.

In this study, we report a biomimetic electronic skin (E‐skin) that closely mimics the structure and functions of natural skin. As shown in Figure 1b, a hierarchical polymeric structure with the top polymer film resembling the relatively dried epidermal skin, whereas the bottom hydrogel layer is akin to fully hydrated dermis tissue. Our strategy to prepare the polymer film from hydrogel surfaces involved a simple yet robust in situ interfacial precipitation polymerization, which could even be extended to real biological dermis, namely, porcine skin. The resulting films comprised of interconnected polymer nanoparticles, analogous to microstructured and interlocked corneocytes in the SC of the epidermis. This layer of porous film is seamlessly connected with the hydrogel through an interface layer that contains a hybrid of hydrogel and polymer nanoparticles, creating a durable coating on the hydrogel substrate. While the polymer film retains exceptional permeability towards small molecules like water, thanks to its hydrophilicity and porous structure, surface hydrophobicity can be tailored through simple chemical modification, effectively preventing excess water evaporation from the hydrogel matrix. In addition to this, our hierarchical polymer system could lead to efficient E‐skin sensors for tactile sensing through a self‐powered triboelectric nanogenerator (TENG).^[^ ^37^, ^38^ ^]^ Meanwhile, this system provided a materials platform to physically integrate a highly sensitive and vertically aligned gold nanowire (v‐AuNW) resistive sensor onto the hydrogel surface (Figure 1c). This v‐AuNW resistive sensor could be integrated as patterned thin “tattoos” by locking it between the two layers via an encapsulation process. The sensor was firmly embedded in the porous polymer film of the hydrogel and exhibited excellent temperature and pressure sensing performance. We believe this biomimetic artificial skin design may simplify and accelerate the development of remote health monitoring, prosthetic skin, and soft robotics.^[^ ^39^, ^40^, ^41^, ^42^, ^43^ ^]^

## 2 Results and Discussion

### 2.1 Preparation of Porous Polymer Films on Hydrogel Surface to Mimic Epidermis‐Dermis Structure of Human Skin

**Figure 2:**
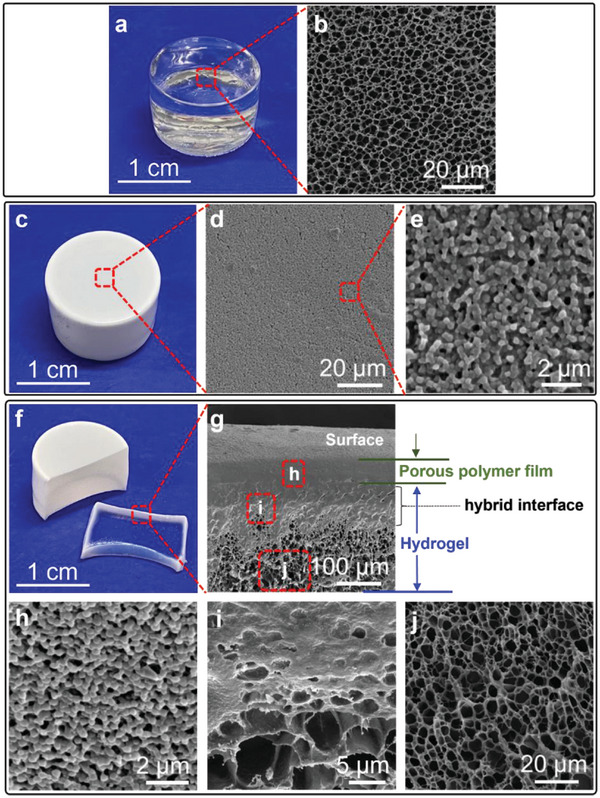
Optical and microscopic characterization of PHPMA‐coated hydrogel. a,b) Optical image and SEM image for surface morphology of blank Agar/PAAm hydrogel; c,d,e) Optical image and SEM images for surface morphology of PHPMA coated hydrogel; f) Optical image of cross‐sectional PHPMA coated hydrogel; g) SEM image for the morphology of cross‐section of PHPMA coated hydrogel, showing a bilayer structure of porous polymer film and hydrogel with a hybrid interface; h,i,j) SEM images for detailed morphologies of three enlarged areas in Figure 2g.

The synthesis employs an in situ interfacial precipitation polymerization approach to grow a porous polymer film from a hydrogel surface, which involves a two‐step soaking‐polymerization process (Figure 1c).^[^ ^44^, ^45^, ^46^, ^47^ ^]^ The radical polymerization is initiated by a redox initiation system, ammonium persulfate (APS)/*N,N,N’,N’*‐tetramethylethylenediamine (TMEDA), in which APS and TMEDA form charge transfer complex and generate hydroxyl (HO·), bisulfate (HSO_4_·) and alkylaminoethyl radicals for radical polymerization within a relatively short time.^[^ ^48^ ^]^ These two components will not generate radicals in the absence of one another at room temperature. The first step involved absorbing APS into the hydrogel which diffused into a certain depth from the surface. Unless otherwise stated, the hydrogel substrate used in this study is an Agar/PAAm double‐network hydrogel which possesses a comparable mechanical strength to human skin (Young's modulus 0.5–1.95 MPa).^[^ ^9^, ^49^ ^]^ Subsequently, the APS‐loaded transparent gel was immersed in a monomer solution containing monomer (HPMA, 2‐hydroxypropyl methacrylate, Figure 1c), crosslinker (MBAA, *N,N’*‐methylenebisacrylamide) and TMEDA. Notably, the key feature of this monomer is its good solubility in water (≈13 w/v% at room temperature for HPMA^[^ ^50^, ^51^ ^]^), but poor solubility of its polymer counterpart. The loaded APS in the gel diffused into the monomer solution due to the concentration gradient. The in situ chain‐growth polymerization occurred at the interface of hydrogel and monomer solution where APS was quickly reduced to generate radicals after coming into contact with TMEDA that subsequently polymerized HPMA. With increasing molecular weight, the solubility of the polymer chains decreased and precipitated into near‐spherical nanoparticles that attached on the hydrogel surface. After 10 min of polymerization, the pristine Agar/PAAm hydrogel changed from transparent (**Figure** **2**a) to opaque with a thin layer of PHPMA film (Figure 2c) after removal from the monomer solution. SEM revealed completely different surface morphologies of the hydrogel before and after polymerization. The opaque coating morphologically presented densely interconnected polymer nanoparticles with an average particle size of 270 nm (Figure 2d,e; Figure S1, Supporting Information) that created nanosized porous channels in the film, whereas the blank hydrogel showed characteristic crosslinked networks with >3 µm macropores (Figure 2b).

It was notable to see that the film was tightly bonded onto the hydrogel and no discernible damage or delamination of the polymer layers was observed after applying mechanical stresses and sheer force on the film. The robustness of the polymer coating suggests the existence of strong adhesion or interactions between the film and hydrogel substrate. The uniform attachment of a thin film was also evident with the cross‐section of the hydrogel sample as illustrated in the optical image in Figure 2f, indicating an opaque polymer film on the surface of the transparent hydrogel substrate. Visualization by fluorescence microscopy under a bright field suggested the thickness of the film is around 140 µm (Figure S2a, Supporting Information). Copolymerizing a fluorescent monomer (fluorescein *O*‐acrylate, FA) with HPMA in the monomer formulation allowed for fluorescent labeling of the film which showed a fluorescent layer that overlapped well with bright field image (Figure S2b, Supporting Information), revealing that the opaque film was indeed created by PHPMA nanoparticles.

The visualization by SEM through the cross‐section of the polymer nanoparticle‐coated hydrogel (Figure 2g) showed gradient morphological structures with varied depths from the surface. Three different morphologies can be roughly identified from the top to the bottom. The top layer (≈0–40 µm thickness) presented a homogeneous film comprising interconnected nanoparticles (Figure 2h). Its morphology close to the surface (Figure 2h) is the same as that on the surface itself, both of which are interconnected nanoparticles with comparable particle sizes (200–300 nm). Deeper from the top layer (40–140 µm), the nanoparticles are progressively smaller but fused together with the presence of big pores (Figure 2i; Figure S3, Supporting Information) due to the co‐existence of polymer nanoparticle and hydrogel network forming a hybrid interface. The top layer and the hybrid area are connected seamlessly with a total thickness of ≈140 µm, which was consistent with the measured thickness from fluorescence microscopy as discussed above. In contrast, the bottom (beyond 140 µm) presenting a porous crosslinked network (Figure 2j) is similar to the control hydrogel.

The hybrid interface is a layer of both PHPMA nanoparticles and hydrogel substrate, which can be demonstrated by measuring the thickness of the top porous polymer film using a surface stylus profilometer. Due to the seamless interface between them, SEM of the hydrogel cross‐section was unable to provide a precise thickness of porous polymer film in the top layer. To circumvent this problem, we grew a patterned flat stage of polymer film on the surface of hydrogel using a mask with a cylindrical hole (4 mm diameter and 5 mm thick) in the middle (Figure S4a, Supporting Information). The thickness of the film could be measured by the height of the stage using a surface profilometer. Patterned film growth was performed using the same two‐step procedure. A white and round shape pattern on the hydrogel surface was produced after removing the mask (Figure S4b, Supporting Information). A single scan along the hydrogel surface covered by the pattern using a stylus in the profilometer provided a height profile of the stylus trace, showing a stage in the middle with a uniform height of ≈38 µm (Figure S4c, Supporting Information), that is., thickness of the top porous polymer film. Compared to the thickness of the entire opaque film (≈140 µm) measured by fluorescence microscopy, it is sufficient to conclude that the hybrid interface had a thickness of ≈102 µm, which is embedded in the hydrogel matrix.

These findings demonstrated that the prepared polymer nanoparticle‐coated hydrogel has a bilayer structure proposed in Figure 1b that is similar to human skin with the top porous polymer film as the epidermis, and bottom hydrogel as the dermis. Particularly, the top layer comprises a porous polymer film with interconnected nanoparticles. The particle size (≈270 nm) is comparable to the thickness of disc‐like corneocytes (up to 1000 nm)^[^ ^52^ ^]^ in the SC, which demonstrates a structural mimicry of the prepared film toward the epidermis. This film is grown from the hydrogel surface and tightly bonded to the hydrogel because of the presence of a hybrid interface. The formation of the interfacial layer is most likely caused by the diffusion of the monomer, crosslinker, and TMEDA into the hydrogel substrate and subsequent precipitation polymerization after coming into contact with APS in the hydrogel. Meanwhile, the diffusion of APS into monomer solution at the hydrogel surface also causes interconnected polymer nanoparticles to form on the interface. This simultaneous two‐way diffusion (or mass transfer) governs the interconnection of porous polymer film and hydrogel substrate with a seamless interface. The interaction between them is most likely chain entanglement, particle coalescence, and/or hydrogen bonding. As a result, the formed porous polymer film was anchored to the hydrogel substrate, which can produce robust, spatially controlled patterns of polymer films with high resolution on the hydrogel surface (Figure S5 and Video S1, Supporting Information).

These results differed from the reports by Raghavan and coworkers,^[^ ^45^, ^46^ ^]^ despite their use of the same redox initiation system (APS/TMEDA). In their reports, multilayer hydrogels instead of polymer nanoparticles were produced on the surface of substrates which were peelable without interconnection between hydrogel layers and the substrate. The main reason is the one‐way diffusion of APS into monomer solution instead of two‐way diffusion. The hydrogel layer formed in situ on the substrate surface is viscous and thick enough to significantly impede the movement of molecules and consequently blocks the reagents in monomer solution from diffusing into the substrate. In our study, the precipitated polymers in situ create nanoporous channels full of free water molecules which allows for backward diffusion of monomers into the hydrogel substrate without slowing down. This hypothesis can be validated by the result of changing the monomer from HMPA to *N,N*‐dimethylacrylamide (DMA) where both monomer and polymer are soluble in water. With the same protocol and monomer concentration, it showed the same results as reported by Raghavan and coworkers that have a transparent, peelable hydrogel layer (thickness ≈1 mm) around the Agar/PAAm substrate (Figure S6, Supporting Information). These findings initially demonstrated our hypothesis of a two‐way diffusion mechanism.

### 2.2 Structural Diversity and Versatility of Polymer Films

Apart from the monomer of HPMA that exhibits excellent precipitation polymerization behavior in aqueous solutions, another monomer, diacetone acrylamide (DAAM, Figure 1c), also suitably produced porous polymer films on hydrogel surfaces. Using the identical formulation and procedure, we grew a thin layer of PDAAM film from the Agar/PAAm hydrogel (Figure S7a, Supporting Information), which was more transparent than that of PHPMA. The surface morphology of the film from SEM presented similarly interconnected nanoparticles as observed in the HPMA system but with a smaller nanoparticle size (≈120 nm) (Figure S7b,c, Supporting Information). The film thickness was also measured on a patterned film by surface profilometry to be ≈30 µm (Figure S8, Supporting Information). Compared to PHPMA films, smaller particle size (120 vs 270 nm) and thinner film layers (30 vs 38 µm) from PDAAM films means that light is scattered less, rendering them more transparent. Another property of PDAAM films compared to those comprising PHPMA is the reactive ketone functionality that can engage in many organic reactions including reduction, oxidation, and addition reactions. One of the more popular addition reactions is to react the carbonyl group with a primary amine to produce an imine, also known as the Schiff base reaction, which is employed for the hydrophobic modification of the PDAAM film (vide infra). Additionally, this monomer has been extensively employed to produce functional polymer nanoparticles with diverse morphologies through a polymerization‐induced self‐assembly process.^[^ ^53^, ^54^ ^]^

The porous polymer films could be grown from many hydrogel substrates with distinct compositions apart from the Agar/PAAm hydrogel. These could be any single and double network hydrogels or any other hydrogel composites. In this study, we selected four other typical hydrogels to demonstrate the substrate versatility, namely, alginate, polyvinyl alcohol (PVA), gelatin and PDMA. Optical images of these hydrogels before and after coating suggested homogeneous coatings and excellent performance of polymer film growth (Figure S9, Supporting Information).

Aside from hydrogel substrates, this porous polymer film could be grown from a biological tissue. A piece of porcine skin (20 × 12 × 6 mm^3^ Figure S10a, Supporting Information) taken from a porcine tenderloin was thus employed as the substrate. To mimic the epidermis structure, a thin top layer of the skin (≈0.5 mm thick) that is regarded as the epidermis of the porcine skin was cut off to expose dermis tissue on the one‐half side of the sample, with the other half remaining intact as the control. It is interesting to observe from SEM that the surface morphology of the skin epidermis presented nanostructure with particle size around 300 nm (Figure S10b, Supporting Information) that are the corneocytes embedded in a lipid matrix as many other mammalian skins have, whereas the epidermis‐off skin surface showed a typical tissue like pattern (Figure S10c, Supporting Information). The whole piece of skin was employed to grow a PHPMA polymer film using our standard two‐step soaking‐polymerization protocol. The epidermis‐off skin appeared to develop an opaque coating on the surface (Figure S10d, Supporting Information). As visualized by SEM, the surface of the epidermis‐off skin after polymerization presented different morphologies compared to the original substrate, presenting aggregated polymer nanoparticles after polymer coating (Figure S10e, Supporting Information) that are very similar to those grown from hydrogels. In contrast, the epidermis‐on skin showed negligible difference except for slight discoloration. This result demonstrated our synthetic method to prepare porous polymer films applies to biological tissues (dermis layer) due to their comparable material environment for interfacial precipitation polymerization and polymer film growth, despite of the presence of fat cells in dermis tissues. More significantly, the polymer film remained intact after being subjected to mechanical stresses (multiple bending and stretching) (Figure S11, Supporting Information).

### 2.3 Fine‐Tuning Hierarchical Structures by Adjusting Film Thickness and Nanoparticle Size

The film thickness could be easily controlled in the synthetic process by varying the polymerization time. We maintained a constant soaking time of 5 min in the APS solution but different polymerization times (8, 10, 15, and 20 min) for film growth. The polymer nanoparticle‐coated hydrogels were analyzed by SEM and profilometry to determine surface morphology, nanoparticle size, and film thickness. As shown in Figure S12 (Supporting Information), the surface morphologies of the films at different polymerization times presented very similar results with interconnected nanoparticles and 270–300 nm particle sizes (Figures S1; S12b–e and S13, Supporting Information). In contrast to unchanged nanoparticle sizes and morphologies with polymerization time, the film thickness measured by surface profilometry increased consistently, with 35, 38, 45, and 58 µm for 8, 10, 15, and 20 min, respectively (Figure S12f, Supporting Information). The results of film thickness with the polymerization time seemed not to follow an obvious mathematical model such as the first‐order model as investigated by Raghavan and coworkers.^[^ ^45^ ^]^ This suggests a complicated mechanism for our process with two‐way mass transfer involved (diffusion of APS initiator into monomer solution and reactants in monomer solution into hydrogel). The film growth may thus include both outwards in the top layer and inwards in the hybrid interface. This mechanistic investigation is currently ongoing and outside of the scope of this study.

Nonetheless, based on these results we can conclude that the film thickness could be controlled up to 58 µm by varying the polymerization time. Unfortunately, extending the polymerization time is not ideal for obtaining thicker coatings, as it will lead to quick drainage of the initiator from the hydrogel and cause excessive swelling of the hydrogel in monomer solutions, thereby changing the mechanical properties of the hydrogel substrate. Therefore, we employed a layer‐by‐layer method by repeating the two‐step process to extend the thickness of polymer films.

**Figure 3:**
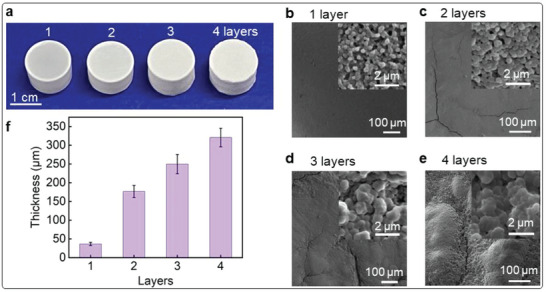
PHPMA‐coated hydrogels with different layers of coating. a–e) Optical image and SEM images for surface morphologies of the hydrogels with 1–4 layers of PHPMA coatings. The insets are SEM images for zoomed‐in surface morphologies; f) the thickness of different layers of PHPMA coating.

After completing the growth of the first layer of polymer film, we repeated the same process to grow the second layer and further to grow the third and fourth layers. It was interesting to find that the films presented increasing roughness and decreasing hydration as each layer was added, which was apparent from their surface appearance (**Figure** **3**a). The surface roughness was also confirmed by SEM, showing a flat surface for the first layer becoming progressively rougher as more layers were added (Figure 3b–e). We also found that the films had some cracks when two or more layers were added after lyophilization, which suggested a less flexible film compared to single‐layered counterparts. Additionally, the nanoparticle sizes changed with the layers from 270 to 860 nm (the insets in Figure 3b–e; Figures S1 and S14, Supporting Information). As expected, the film thickness increased significantly with each layer, from 40 to 330 µm (Figure 3f).

Despite the layers being prepared iteratively, there are no discernible interfaces between them, as illustrated by the SEM image of the cross‐section of the coated hydrogel with four layers of coating (Figure S15, Supporting Information). While soaking in excess water did cause the coated hydrogels to swell (Figure S16a,b, Supporting Information), but there was no observed delamination or disintegration of the polymer coating from the hydrogel substrate (Figure S16c and Video S2, Supporting Information), highlighting the robust bonding between individual polymer layers and between the polymer layers and the hydrogel substrate. This is attributed to nanoparticle assembly and porous structure of the film, which allows for the diffusion of monomers into the lower layers and polymerizing to form nanoparticles in the pores. This film growth mechanism enabled the seamless interconnection between layers even layer‐by‐layer procedures were employed. This layer‐by‐layer synthetic process can also be applied to the monomer DAAM as well, which presents a thinner coating (up to 245 µm), a flatter surface, and a smaller nanoparticle size (up to 400 nm) (Figure S17, Supporting Information) compared to PHPMA counterparts.

### 2.4 Water Loss Prevention Property

Having confirmed the structure of our hydrogel artificial skin, we next investigated its functions for diverse applications. One of the primary functions of human skin is protection from excess water loss of the human body. In our study, we sought to demonstrate similar functions of our porous polymer coatings on hydrogel surfaces, which can control the dehydration of the hydrogel through a simple modification of the surface coating. We found that the surface coatings of PHPMA on hydrogels did not effectively prevent water evaporation even if a thick layer coating (four layers, 330 µm) was applied (59 wt.% for PHPMA coated hydrogel vs 70 wt.% for the blank gel at 24 °C and 30% relative humidity, Figure S18, Supporting Information). This is attributed to the film's intrinsic hydrophilicity and porosity that is unable to impede the movement and evaporation of water molecules through it. The polymer films are still relatively hydrophilic due to the presence of hydroxyl groups in PHPMA polymer chains, despite the nanoparticles being insoluble in water. This is similar to corneocytes in the SC layer of human skin, which are typically supplemented by extracellular lipids to prevent water loss. The extracellular lipids render the epidermal layer hydrophobic and thus prevent free penetration and diffusion of water molecules.

Inspired by this fact, we thought to prevent water loss by filling the nanopores in the film with a lipid, acting as a moisturizer of sorts. There are several reports of a similar concept using a double hydrophobic coating strategy^[^ ^55^, ^56^ ^]^ in which the first layer coating (aliphatic compounds) encapsulates the hydrogel and the second layer (oil), through physical absorption, further prevents the hydrogels from drying. Despite their efficacy, physical absorption is typically less robust and consequently limited in practical uses. In a similar vein, we demonstrated the feasibility of casting an aliphatic amine (dodecylamine, DDA) on the surface of PHPMA‐coated hydrogel through physical absorption. DDA has a melting point of 28–32 °C and was therefore coated onto the gel at an elevated temperature (45 °C). Upon cooling, the coating turned into a solid film after gentle washing in dichloromethane (DCM). Gravimetric analysis exhibited 21 wt.% water loss after 24 h (at 24 °C and 30% relative humidity) compared to 70 wt.% water loss of a blank gel control (Figure S18, Supporting Information). Again, despite the efficacy of physical absorption in this case, it is often difficult to quantitatively control the thickness of a physical coating reproducibly and therefore water loss kinetics. To this end, we sought to use a chemical coating strategy^[^ ^57^, ^58^ ^]^ for our study.

**Figure 4:**
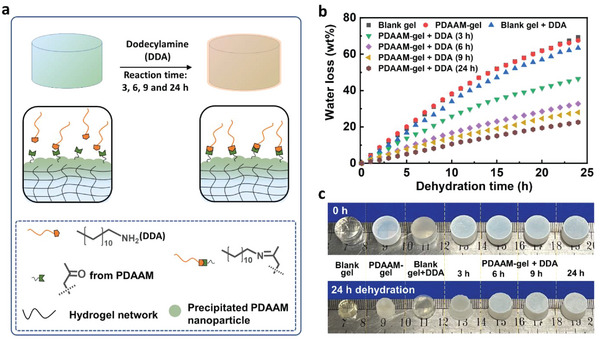
Water loss control of PDAAM coated Agar/PAAm hydrogels chemically treated by dodecylamine (DDA). a) Schiff‐base chemistry between DDA and ketone functionality of PDAAM films on hydrogel surfaces. b) Anti‐dehydration performance. “Blank gel” is the Agar/PAAm hydrogel without coating and DDA treatment. “PDAAM‐gel” is the PDAAM‐coated hydrogel with a 95 µm coating. “Blank gel + DDA” means the sample of blank hydrogel without coating after 24 h curing with DDA. Similarly, “PDAAM‐gel + DDA” means the sample of PDAAM‐coated hydrogel with a 95 µm coating cured with DDA at different times (3, 6, 9, and 24 h). c) Optical images for the control and treated hydrogels after 0 h and 24 h dehydration. The samples are in the same order as those in Figure 4b, from blank gel to PDAAM‐coated hydrogel after 24 h curing with DDA.

We employed the PDAAM‐coated hydrogel (Agar/PAAm) as an illustrative example by taking advantage of the ease of its chemical modification. The surface hydrophobicity was tailored by reacting DDA with the ketone functionality of the DAAM unit via a Schiff‐base reaction (**Figure** **4**a). The increased hydrophobicity of the hydrogel surface was expected to prevent water evaporation from the hydrogel. Specifically, four PDAAM‐coated hydrogels with a constant film thickness of 95 µm were immersed into liquid DDA at 45 °C. After 30 s soaking, the gels were wrapped with a thick layer of oil. Next, the gels were cured for different time periods (3, 6, 9, and 24 h) at room temperature, respectively. After curing, the gels were washed by DCM to remove excess unreacted DDA. The dried gels showed negligible change in appearance except being slightly opaquer than the unmodified PDAAM coated gel (Figure 4c). After 24 h dehydration, the gels exhibited a different extent of water loss and different sizes across four samples and three control gels (Figures 4b,c), which demonstrated tunable water loss from 20 wt.% (24 h curing time) to 70 wt.% (control sample without DDA treatment). Longer DDA curing times of PDAAM films indeed translated to better protection from water evaporation due to more hydrophobic chains being incorporated into the films.

### 2.5 Self‐Powered Triboelectric Nanogenerator (TENG) for Pressure Sensing

**Figure 5:**
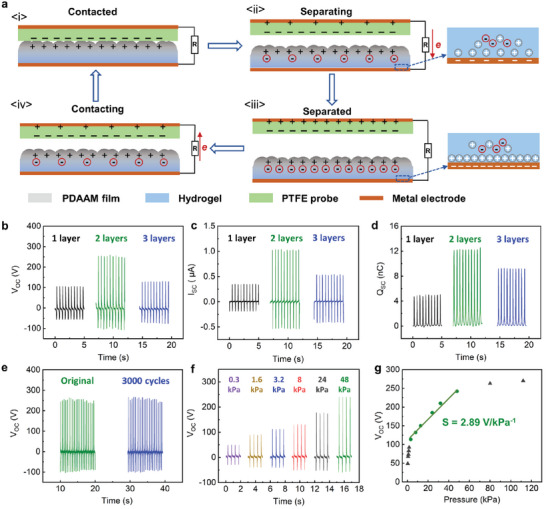
TENG of PDAAM coated hydrogel (PDAAM/hydrogel‐TENG). a) working principle of TENG device in the contact‐separation mode. The output performance of PDAAM/hydrogel‐TENG with different PDAAM thickness (1 layer for 30 µm, 2 layers for 95 µm and 3 layers for 170 µm) under an applied pressure of 80 kPa in *V*_OC_ b), *I*_SC_ c) and *Q*_SC_ d). e) Stability test of the TENG device. f) The illustrative *V*_OC_ signals under various pressures. g) The plot of *V*_OC_ versus pressure.

Similar to human skin, our biomimetic hierarchical polymeric hydrogels can enable triboelectric energy generation via simple touch, which can be used for designing self‐powered pressure sensors. To demonstrate this, we employed polytetrafluoroethylene (PTFE) as the triboelectrically negative probe due to its high tendency to accumulate negative charges compared to the polymer (PDAAM in this case). **Figure** **5**a illustrates the possible working mechanism in contact‐separation mode, similar to the other literature related to hydrogel‐based triboelectric nanogenerators.^[^ ^38^ ^]^ Once PTFE comes into contact with the PDAAM layer of the TENG, triboelectrification occurs at the interface due to their different electronegativities. As a result, equivalent but opposite charges are generated on the surfaces of PTFE and the PDAAM film (as shown in Figures 5a, i). After separating these two materials, ions in the hydrogel will move to achieve a balance of static charges on the insulating PDAAM layer (Figure 5a,ii), resulting in the formation of an excess ion layer at the PDAAM/hydrogel interface. Simultaneously, the electric double layer formed at the metal wire/hydrogel interface becomes polarized, leading to the creation of an equal number of excess positive charges at the interface. A negative charge will flow from the PTFE layer to the metal wire/hydrogel interface along the external circuit until all static charges are balanced, generating a flow current until two materials are completely separated (Figure 5a,iii). When the PTFE makes contact again with the PDAAM film, the whole process will be reversed, and the negative charge will flow back to the PTFE (Figure 5a, iv). When continuously repeating the contact–separation motions, a periodic electrical current will be generated as shown in Figure 5b. The performance is determined by the output voltage or current signals.

It is worth to note that hydrogel‐based hybrid materials have been recently developed to be used for various soft electronic devices including strain sensors.^[^ ^59^, ^60^ ^]^ Polymer films, particularly PDMS and other silicone rubbers, are commonly employed as triboelectric materials when fabricating these devices. However, the mechanical mismatch and weak bonding interface between the hydrophobic elastomers and hydrophilic hydrogels leads to a deterioration in charge induction and consequently the overall performance of the TENG devices.^[^ ^61^ ^]^

To overcome this, conventional methods used for fabricating hydrogel‐based TENGs with tough interfacial bonding between elastomers and hydrogels typically involve energy‐intensive procedures.^[^ ^62^ ^]^ In this study, however, the TENG device comprised of a single side PDAAM coated conductive hydrogel (PDAAM/hydrogel‐TENG) which was prepared in situ through a simple two‐step soaking‐polymerization process. In this system, the top PDAAM film served as the dielectric layer while the underlying hydrogel (with 8 m LiCl) served as the ion‐conducting electrode. More importantly, the nanoporous structure of PDAAM increased the surface roughness and therefore triboelectric effect. It is also noted that the added LiCl in hydrogel can effectively prevent water loss during measurement.^[^ ^63^, ^64^ ^]^

The output of a 2.5 × 2.5 cm^2^ PDAAM/hydrogel‐TENG device (Figure S19, Supporting Information) at a fixed contact–separation frequency was measured with samples varying in polymer film thicknesses at different layers. Figure 5b,c,d show the performance of PDAAM/hydrogel‐TENG at three different PDAAM layer thickness (1 layer for 30 µm, 2 layers for 95 µm, and 3 layers for 170 µm). It can be seen that the open‐circuit voltage (*V*_OC_) and short‐circuit current (*I*_SC_) were greatly enhanced by almost 2.5 times as the thickness of polymer films increased from 30 µm (*V*_OC_ = 100 V, *I*_SC_ = 0.35 µA) to 95 µm (*V*_OC_ = 251 V, *I*_SC_ = 1.05 µA) under an applied pressure of 80 kPa. The enhanced output performance might be attributed to the unique porous structure of PDAAM film. As we demonstrated previously, the film is still hydrophilic despite the polymer is insoluble in water. As such, water in hydrogel could infiltrate into the polymer film to a certain extent. If the polymer film is as thin as 30 µm, the infiltrated water may interfere with the electrostatic induction in the film, whereas the thicker trilayer at 95 µm has less interference and consequently higher output performance. However, a further increase of PDAAM layer thickness to 170 µm led to the decline of *V*_OC_ and *I*_SC_ (110 V and 0.42 µA, respectively). This is because excessively thick tribolayers tend to have high electrical resistance, which can impede the flow of charges through the PDAAM layer, thus leading to a decrease in the output current. Accordingly, the short‐circuit charge (*Q*_SC_) of different thickness TENG showed a similar trend as *V*_OC_ and *I*_SC_ (Figure 5d). The device with 95 µm thickness of the PDAAM layer presented the highest *Q*_SC_ of 12.1 nC.

We further demonstrated the durability of the as‐prepared triboelectric energy generator by continuous contact‐separation testing on the 95 µm PDAAM‐coated hydrogel. Figure 5e shows that the device exhibited a consistently stable output throughout 3000 cycles of contacts. Unlike conventional hydrogel–elastomer based devices that often experience a decline in performance over prolonged usage due to their weak interfacial bonding strength between hydrogels and elastomers,^[^ ^62^ ^]^ the seamless bonding interface in our PDAAM/hydrogel‐ highlights the exceptional durability and robustness for long‐term usage.

We further explore our triboelectric devices for self‐powered pressure sensing applications. Figure 5f shows the stable signals in responses to various applied pressures (from 0.3 to 48 kPa), indicating increased output voltages with the increased pressure. It is interesting to observe a linear plot of *V*_OC_ with pressure (Figure 5g) in the range of 3.2 to 48 kPa which sits within the common range of pressure in our daily life. The pressure sensitivity was estimated to be 2.89 V/kPa^−1^ in this pressure range (represented by an output voltage as a function of the applied pressure), which is much higher than those previously reported triboelectric‐based sensory FEP/ITO and gold nanowire systems.^[^ ^65^, ^66^ ^]^

Furthermore, a body motion sensor was designed for detecting various movements of the human body. In this design, the PDAAM/hydrogel‐TENG was assembled using PTFE as the triboelectric layers, PDMS as the spacer, and a small backside metal plate as the electrode (Figure S20a, Supporting Information). The bending motion will lead to the contacts between the PTFE and PDAAM, hence generating an output voltage. We securely fixed both ends of the as‐fabricated TENG device to the volunteer's finger, wrist, and elbow, and the sensor could transduce mechanical motions to real‐time voltage signals. As shown in Figure S20b,c,d (Supporting Information), the device can generate distinctive *I*_SC_ signals corresponding to the degree of various body bending actions. Among all the signals collected from these motions, those from wrist bending are the largest, indicating the greatest forces applied to the sensing device. This proof‐of‐concept demonstrates the potential of our device for self‐powered daily and clinical body motion monitoring.

### 2.6 Built‐In Gold Nanowire Sensors for Pressure and Temperature Sensing

Inspired by the unique structure of human skin with its various sensory receptors in the epidermis and dermis (Figure 1a), we have developed an encapsulation process that enabled seamless integration of gold nanowire‐based resistive sensors into the hierarchical polymeric system to impart a sensing functionality. To achieve this, we took advantage of the in situ polymer film growth and capability of patterning to develop a rapid process for the integration of a hydrogel with an ultrasensitive and multifunctional E‐skin sensors that consisted of the vertically aligned gold nanowire (v‐AuNW) (“tattoo”) supported by a styrene‐ethylene‐butylene‐styrene (SEBS) elastomer.

**Figure 6:**
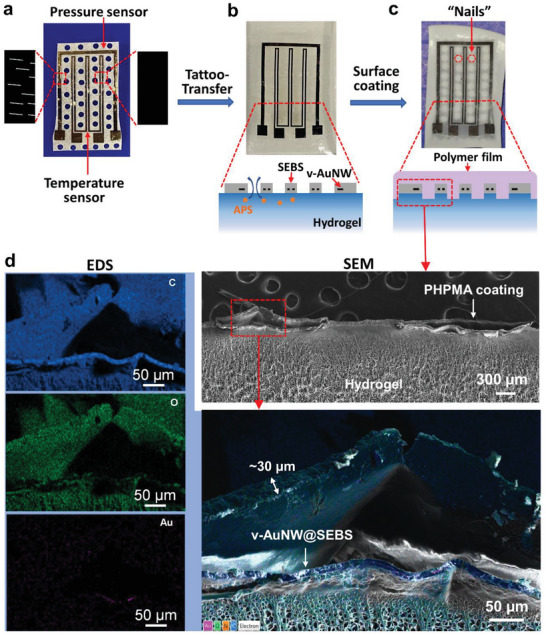
v‐AuNW@SEBS sensor transfer (“tattoo‐transfer”) and integration into hydrogel by porous polymer films and the structural characterization of the integrated device. a–c) Digital photos of free v‐AuNW@SEBS sensor with two sensing patterns and punched holes a), transferred free sensor on hydrogel surface b), and the integrated device with v‐AuNW@SEBS sensor on hydrogel being physically locked by a porous PHPMA film c). d) SEM images and EDS maping (C, O, and Au elements) of a cross‐section of the integrated device for structural confirmation.

The v‐AuNW@SEBS sensor was first prepared by printing the v‐AuNW in SEBS thin film (thickness ≈10 µm) and followed by encapsulating with another layer of SEBS thin film (thickness ≈10 µm) but with line connectors exposed. The v‐AuNW sensor was designed with two patterned circuits (**Figure** **6**a), the outer ring with aligned cracks for pressure sensing and the inner ring without cracks for temperature sensing. The free v‐AuNW@SEBS sensor was sitting on a PVA supporter, which was punched manually with distributed holes (≈1.5 mm diameter) in the area of free v‐AuNW as shown in Figure 6a. This free sensor was transferred to a neat hydrogel (Agar/PAAm, 20 × 15 × 2 mm^3^) surface by using water to dissolve the PVA supporter, consequently exposing the whole v‐AuNW@SEBS sensor (Figure 6b). The hydrogel was then carefully immersed in APS solution to load the radical initiator and followed by monomer solution to grow a layer of porous polymer film (Figure 6c). To expose the line connectors, the area was covered by a thin layer of PDMS to prevent polymer growth.

The obtained sensor device suggested a layer of opaque polymer film on the top of v‐AuNW@SEBS sensor (Figure 6c) which physically locked the sensor into the hydrogel presenting excellent structural integrity and robustness. Water flushing on the surface of the hydrogel did not cause delamination of the sensor from the hydrogel substrate, suggesting strong bonding between them (Video S3, Supporting Information) and successful integration. By contrast, the sensor before polymer growth could be easily washed away by water flushing due to the weak interfacial interaction between hydrogel and SEBS. Most significantly, on the surface of the hydrogel and sensor, the area with punched holes exhibited obvious polymer growth with more opaque films, whereas the area that was covered with SEBS was far more transparent. It indicated that the loaded APS initiator diffused from the hydrogel substrate to the interface through the punched holes, not SEBS, and reacted with TMEDA to generate radicals followed by polymerizing monomers to form polymer nanoparticles. The polymer film grew along the edge of the SEBS film and gradually encapsulated the whole sensor in the film. The polymer films grown from the holes served as “nails” to physically lock hydrogel and SEBS. Compared to traditional methods using adhesives or chemical treatment of elastomer surface, this encapsulation approach is non‐invasive toward the locked components, which allows for potential recycling of embedded sensors and replacement of the host hydrogel part after use. Meanwhile, the embedded sensor could be assembled in a modular approach that enables access to encapsulated and complex electronics as a single piece of object before integration into hydrogel.

A “nails‐like” structure was demonstrated by morphological characterization. As shown in Figure 6d, the cross‐section of the integrated sensor on the hydrogel surface was visualized by SEM, which clearly showed the hydrogel substrate, v‐AuNW@SEBS sensor, and top polymer film. The EDS mapping indicated the location of SEBS film and AuNW. The polymers that grew out of the holes had a tight connection with the top film, whereas SEBS films were covered by a polymer film with a thickness of ≈30 µm. Due to the incompatibility of SEBS, the hydrogel, and its polymer films, SEBS films delaminated from the hydrogel after lyophilization due to shrinkage of the hydrogel.

**Figure 7:**
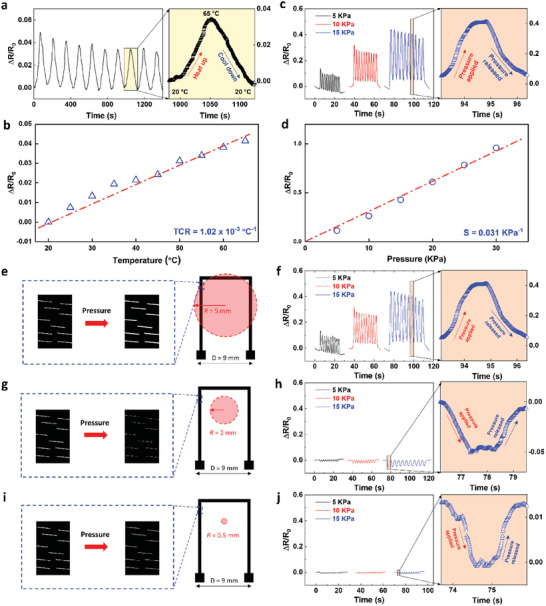
Sensing performance of the integrated hydrogel sensor device (artificial fingertip) toward temperature and pressure. a) Cyclic temperature sensing performance of the artificial fingertip during 10 heating‐cooling cycles between 20–65 °C. b) Plot and linear fit of normalized resistance changes against the temperature of the artificial fingertip. c) Cyclic pressure sensing performance of the artificial fingertip during 10 loading‐unloading pressure cycles at different pressures (5, 10, and 15 kPa). d) Plot and linear fit of normalized resistance change against the pressure of the artificial fingertip. e) Schematic of the application of a blunt probe (5 mm radius) on the sensor, inducing crack propagation. The magnified insert shows how the cracks in the v‐AuNW sensor changed with pressure being applied. f) Cyclic pressure sensing performance of the artificial fingertip during 10 continuous pressure cycles at 5, 10, and 15 kPa with a blunt probe. g) Schematic of the application of a medium size probe (2 mm radius) on the sensor. The magnified inset shows how the cracks of the v‐AuNW sensor changed with pressure being applied. h) Cyclic pressure sensing performance of the artificial fingertip during 10 continuous pressure cycles at 5, 10, and 15 kPa with a medium size probe. i) Schematic of the application of a sharp probe (0.5 mm radius) on the sensor. The magnified inset shows how the cracks of the v‐AuNW sensor changed with pressure being applied. j) Cyclic pressure sensing performance of the artificial fingertip during 10 continuous pressure cycles at 5, 10, and 15 kPa with a sharp probe.

Next, we evaluated the sensing performance of the embedded E‐skin sensors toward temperature and pressure mimicking the function of a human fingertip. The outer ring sensor was sensitive to pressure due to the introduction of programmed cracks,^[^ ^67^ ^]^ which was thus used for pressure sensing tests. The inner ring sensor, however, had no such cracks and thus functioned as a soft resistive temperature sensor.^[^ ^67^ ^]^ **Figure** **7** illustrates the sensing responses of the artificial fingertip (integrated sensor on hydrogel surface) toward temperature and pressure variations. The electrical resistance increased linearly with temperature increasing from 20 to 65 °C (Figure 7a). The temperature coefficient of resistance (TCR) was estimated by *TCR* = Δ*R*/(*R*_0_Δ*T*), where *R*_0_ is the original resistance of the sensor and Δ*R* is the resistance change corresponding to the temperature change (Δ*T*). The TCR of our sensor is 1.02 × 10^−3^ °C^−1^ (Figure 7b), comparable to the bulk v‐AuNW sensors in the previous report.^[^ ^67^ ^]^ Different pressures ranging from small to gentle touch (5–30 kPa) gave different resistance changes of the artificial fingertip, indicating a linear relationship of electric resistance change (Δ*R*/*R*_0_) as a function of pressure (Figure 7c,d). The sensitivity of the pressure sensor could be defined as S = Δ*R*/(*R*_0_ *P*), where *P* is the pressure applied. The pressure sensor showed an excellent sensitivity S = 0.031 kPa^−1^ at the giving pressure range. Meanwhile, this sensory system presented excellent stability and durability as demonstrated by the pressure sensing performance with 500 cycles of loading‐unloading at 10 kPa (Figure S21, Supporting Information), although a slight decay was observed in the first 50 cycles.

Remarkably, the embedded sensor was sensitive to the pressing probe with varied sharpness, and its working principle is shown in Figures 7e,g,i. Blunt probe (5 mm radius) provided an increase in resistance, with 11% at 5 kPa pressure, 23% at 10 kPa, and 44% at 15 kPa (Figure 7f). This is mainly attributed to crack propagation induced by stretching when directly pressing the sensor trace line (Figure 7e). Interestingly, resistance decrease was observed when sharper probes (2‐ and 0.5‐mm radius) were applied (Figure 7g,h). The non‐direct contact pressure at the center area resulted in compressional strain applied at perpendicular direction leading to the closure of cracks (Figure 7g,i) and the consequent decrease of overall resistance. However, the relative resistance changes (or sensitivities) were lower for sharper probes than blunt probes due to less deformation caused by far‐away pressing sites.

## 3 Conclusion

We have developed a polymer nanoparticle‐coated hydrogel system inspired by the hierarchical structure and functions of human skin. We then demonstrated that this could be used as a high‐performance E‐skin. The bilayer hydrogel system comprising of a porous polymer film on the hydrogel surface was fabricated by a simple yet robust in situ interfacial precipitation polymerization that involves a two‐step soaking‐polymerization process. The film, with a controllable thickness of 20 to 330 µm, comprised of interconnected polymer nanoparticles, mimicking the nanostructured interlocked corneocytes in the outermost stratum corneum (SC) layer of the epidermis. This synthetic approach achieved a strong interfacial bonding, through a hybrid interface between the nanoparticles and the hydrogel, analogous to the epidermis‐dermis junction. Our methodology also demonstrated broad applicability, as it could be extended to various hydrogel substrates with different chemical compositions and even biological tissue samples, such as porcine skin dermis. Moreover, we successfully integrated a v‐AuNW (vertical gold nanowire) sensor into the interlayer of the hierarchical polymer via a seamless encapsulation process.

The structural similarity of the system to the epidermis‐dermis also translated to a functional similarity, specifically in terms of permeation, protection, and sensing capabilities. The porous polymer film exhibited excellent permeability toward to small molecules including water, which is evidenced by the diffusion of water, monomers, and other reactants in the monomer solution into hydrogel to form hybrid interface during polymerization. By modifying the polymer film's surface hydrophobicity, we could effectively control water loss from the hydrogel substrate. Moreover, the polymer nanoparticle‐coated hydrogel could be utilized to fabricate self‐powered E‐skin for pressure sensors through triboelectrification, displaying high sensitivity. Additionally, we integrated a v‐AuNW component into the interlayer of porous polymer film and hydrogel to enable temperature and pressure dual sensing functionalities. We believe that our hierarchical polymeric design may represent a powerful strategy to design artificial skins rivaling structures and functions of human skins for broad applications in wearable and implantable bioelectronics, prosthetics, and soft robotics.

## 4 Experimental Section

### Preparation of PHPMA and PDAAM‐Coated Hydrogels

First, a cylindrical agar/PAAm hydrogel (a dimension of 14 mm diameter × 9 mm height) was soaked in an aqueous solution containing APS (10 mL, 30 mg mL^−1^) for 5 min. This was then rinsed with distilled water three times and the excess was pat‐dried. The gel was then immersed in a solution of HPMA (1 m), MBAA (0.02 m), and TMEDA (0.13 m) in water (8 mL). After the appropriate immersion time (typically 10 min), an opaque coating formed around the gel. The polymerization was stopped by transferring the coated gels into distilled water (≈100 mL). The resulting gel was washed with distilled water three times and pat‐dried to remove excess surface water. For the preparation of multilayer coated gels, the aforementioned process was repeated by soaking the gel in APS solution and submerging the initiator‐loaded gel into the HPMA precursor solution, respectively. A variation of this recipe by changing HPMA to DAAM was used to make the PDAAM‐coated gels under identical conditions. To permit image analysis using fluorescence microscopy, the coating polymerization reaction was conducted with the same total monomer concentration (1 m), but with a trace quantity of FA added to the solution ([HPMA]:[FA] = 1000:1). Unless otherwise stated, all reactions were performed at room temperature.

The shape of the hydrogel will affect the uniformity of APS initiator diffusion depth and the subsequent coating layer thickness. For instance, the edge of the cylindrical hydrogel absorbs more APS due to the higher exposure area which would cause a thicker layer coating in the area compared to other flat area after polymerization. However, the shape does not influence film morphology. Meanwhile, the cylindrical hydrogel settled to the bottom of the vial over the course of the polymerization. This container contact did not affect the growth of polymer film either, due to adequate space for the diffusion of APS and monomers.

To coat PHPMA onto different hydrogel substrates (alginate, PVA, PDMA, and gelatin), the synthetic procedures are identical except for the substrates.

### Polymer Film Growth on Porcine Skin

A fresh porcine skin sample (pork tenderloin, 20 × 12 × 6 mm^3^) with the reservation down to the fat layer was cut using a razor blade to remove the epidermis layer (≈0.5 mm). Then, similar to the hydrogel coating procedures, the porcine sample was immersed in APS solution for 5 min and submerged in the HPMA precursor solution for 10 min. The sample was washed with distilled water three times and pat‐dried to remove excess surface water for subsequent SEM analysis.

### Chemical Modification of PDAAM‐coated Hydrogel with Dodecylamine (DDA)

First, DDA (6 g) was melted at 45°C. The DAAM‐coated hydrogel with a 95 µm coating was then immersed into the solution. After 30 s, the gel was taken out and left in a sealed 24 mL vial unperturbed for different time periods (3, 6, 9, and 24 h) at room temperature. After the reaction, the gel was washed three times with DCM to remove the unreacted DDA. The collected gels were subjected to water loss testing.

### TENG Measurement of PDAAM‐Coated Hydrogel

A linear motor (Zaber LSQ450B‐T3A) was employed to provide a recurring back‐and‐forth motion. The distance and frequency of this motion were regulated through the Zaber console software. For all the measurements of energy generation of the hydrogel devices, except the electrical output responses to various applied pressures, the pressure (70 kPa) and frequency (2 Hz) of the step motor were fixed. A force gauge (Mark‐10, M5‐200) was used to measure the dynamic force generated by the linear motor. The current output was measured using a low‐noise current preamplifier (SR570, Stanford Research System, impedance = 4 Ω), and the open circuit voltage was measured by a digital oscilloscope (Rigol DS1074Z).

### Integration of v‐AuNW Sensor into Hydrogel using Porous Polymer Film

The as‐prepared free v‐AuNW@SEBS sensor (the fabrication of the free sensor refers to Supporting Information) was transferred onto a Agar/PAAm hydrogel (20 × 15 × 2 mm^3^) with the SEBS layer in contact with hydrogel surface and water‐soluble PVA layer on top. To expose the whole v‐AuNW@SEBS sensor, the PVA support was removed by carefully dripping DI water until fully swelling and subsequently being wiped with Kimwipe. The line connectors of the v‐AuNW@SEBS sensor were covered by a thin layer of PDMS (200 µm). The whole hydrogel was then soaked in APS solution (30 mg mL^−1^) for 5 min followed by immersing in monomer solution (1 M HPMA, 0.02 M MBAA, and 0.13 M TMEDA) for 10 min to grow a layer of porous polymer film. After the coating process, the protective PDMS layer was carefully removed from the resultant electronic device that was fully washed with DI water to remove the unreacted chemicals. After drying in the air for 10 min, the electronic device was subjected for the measurement of sensing performance.

## Conflict of Interest

The authors declare no conflict of interest.

## Supporting information

Supporting Information

Supplemental Video 1

Supplemental Video 2

Supplemental Video 3

## Data Availability

Research data are not shared.
