# Supplementary material for: Biomimetic Electronic Skin through Hierarchical Polymer Structural Design
Source: Adv Sci (Weinh). 2023 Dec 10;11(7):2309006. doi: 10.1002/advs.202309006 (PMC10870077; doi:10.1002/advs.202309006)
Supplement: Supplementary file 1 — Supporting Information [file ADVS-11-2309006-s003.pdf]

ADVANCED SCIENCE
Open Access

Supporting Information

for *Adv. Sci.*, DOI 10.1002/advs.202309006

Biomimetic Electronic Skin through Hierarchical Polymer Structural Design

Mengnan Zhang, Shu Gong, Karen Hakobyan, Ziyan Gao, Zeyu Shao, Shuhua Peng, Shuying Wu, Xiaojing Hao, Zhen Jiang, Edgar H. Wong, Kang Liang, Chun H. Wang, Wenlong Cheng and Jiangtao Xu**

Supplementary Information

Biomimetic Electronic Skin through Hierarchical Polymer Structural Design

Mengnan Zhang,[1] *Shu Gong,*[2] *Karen Hakobyan,*[1] *Ziyan Gao,*[3] *Zeyu Shao,*[1] *Shuhua Peng,*[3] *Shuying Wu,*[4] *Xiaojing Hao,*[5] *Zhen Jiang,*[6] *Edgar H. Wong,*[1] *Kang Liang*[7] *Chun H. Wang,*[3] *Wenlong Cheng*[2]* *and Jiangtao Xu*[1]*

[1] Centre for Advanced Macromolecular Design and Australian Centre for NanoMedicine, School of Chemical Engineering, UNSW Sydney, NSW 2052, Australia

[2] Department of Chemical & Biological Engineering, Monash University, Clayton, Victoria 3800, Australia

[3] School of Mechanical and Manufacturing Engineering, UNSW Sydney, NSW 2052, Australia

[4] School of Engineering, Macquarie University, Sydney, NSW 2109, Australia

[5] Australian Centre for Advanced Photovoltaics, School of Photovoltaic and Renewable Energy Engineering, UNSW Sydney, NSW 2052, Australia

[6] School of Mechanical, Materials and Mechatronic Engineering, University of Wollongong, NSW 2522, Australia

[7] Graduate School of Biomedical Engineering, UNSW Sydney, NSW 2052, Australia

Corresponding authors: j.xu@unsw.edu.au; wenlong.cheng@monash.edu

Keywords: polymer nanoparticles, hydrogel, interfacial precipitation polymerization, artificial skin, epidermis, electronic skin, sensor

Experimental Section

1.1 Materials

Materials for hydrogel synthesis, polymer coating and characterization: diacetone acrylamide (DAAM, ≥ 99%), acrylamide (AAm, ≥ 99%), *N,N'*-methylenebisacrylamide (MBAA, ≥ 99.5%)， *N,N,N',N'*-tetramethylethylenediamine (TMEDA, ≥ 99%), fluorescein *O*-acrylate (FA, ≥ 95%), ammonium persulfate (APS, ≥ 98%), potassium persulfate (KPS, ≥ 99%), dodecylamine (DDA, ≥ 98%), polyvinyl alcohol (PVA, M_w = 86,000-126,000 g/mol, >99% hydrolysis), sodium alginate and gelatin were all purchased from Sigma-Aldrich. Agar (BR grade) was obtained from Fischer Technical Company. 2-Hydroxypropyl methacrylate (HPMA, ≥ 97%) and *N,N*-dimethylacrylamide (DMA, ≥ 99%) were purchased from Sigma-Aldrich and passed over a short column of basic alumina to remove the inhibitor before use. Dichloromethane (DCM) was purchased from Ajax Chemical and used as received. Ultrapure water was obtained by a Sartorius Arium 611 VF Water Purifier with resistivity up to 18.2 MΩ ·m. All chemicals were used as received unless otherwise indicated.

Materials for sensor assembly: Gold (III) chloride trihydrate, 4-mercaptobenzoic acid, (3-aminopropyl)triethoxysilane, sodium citrate tribasic dihydrate (99.0%), hydrogen peroxide, ammonium hydroxide, *L*-ascorbic acid, liquid metal (Gallium-Indium eutectic), and ethanol (analytical grade), styrene- ethylene-butylene- styrene (SEBS) elastomer and toluene were purchased from Sigma-Aldrich. Positive photoresist AZ 1512 was received from Microchemicals GmbH. Bare silicon wafer <100> was purchased from Electronics and Materials Corporation Limited. All solutions were prepared using Millipore Milli-Q water (resistivity >18 MΩ·cm^{-1}). All chemicals were used as received unless otherwise indicated. Conductive thread was purchased from Adafruit. Water-soluble tape was purchased from Aquasol (ASWT-2).

1.2 Methods

Characterization. Scanning electron microscopy (SEM) images of surface coated hydrogels and porcine skin were collected on a FEI Nova NanoSEM 230 at an accelerating potential of 5.0 kV. For sample preparation, the polymer coated hydrogels were cut to specific shapes and lyophilyzed for 24 h. The lyophilized samples were fixed to a SEM mount and sputtered with 10 nm platinum before measurement. For detailed information on the polymer particle size

(mean diameter/ standard deviation and particle size distribution), at least 200 particles were evaluated for each sample. All Optical photographs were taken by an Apple smartphone (iPhone 11). Fluorescence microscopy was performed on fluorescently labelled samples which were prepared by copolymerising HMPA with a trace quantity of fluorescein *O*-acrylate (FA) ([HPMA]: [FA] = 1000:1). Samples were prepared by cutting 1 mm thick cross-sections of the polymer coated hydrogels. After immersing for 30 min in pure water to remove unreacted fluorescent monomer, the prepared samples were viewed. Fluorescence micrographs were captured on an Olympus IX53 microscope (excitation 488 nm/emission 492-557 nm) with a 4×objective after sectioning the coated hydrogels. The bright field images were also captured to compared with fluorescence results. The obtained fluorescence micrographs were processed using ImageJ software.

Preparation of agar/PAAm hydrogel. Briefly, Aam (180 mg) and agar (20 mg) were mixed with DI water (879 µL) in a 4 mL vial under magnetic stirring for 10 min at 90 °C to form a transparent solution. Stock solutions of KPS (70 µL, 5 mg/mL) and MBAA (39 µL, 10 mg/mL) were then added into the pre-gel mixture. The mixture was incubated in a 70 °C oven for 5 h. The resultant agar/PAAm hydrogel was removed from the vial and has a cylindrical shape with a dimension of 14 mm diameter × 9 mm height.

Preparation of alginate hydrogel. The beadlike hydrogel was prepared by dissolving sodium alginate (5.0% w/v) in DI water. The mixture was then added into an aqueous solution of $CaCl_2$ (0.5 M) dropwise using a transfer pipette. The prepared hydrogel beads were incubated in the solution for 6 h and then washed with DI water 3 times. Gel spheres were then obtained (diameter = ~5 mm).

Preparation of PVA hydrogel. PVA (20 wt%) was dissolved in DI water at 95 °C under vigorous stirring to get a transparent PVA solution. The solution was then transferred into a 4 mL vial and cooled at -20 °C for 1 h. Afterward, the resulting gel was subjected to a thawing treatment for 12 h to form a physically cross-linked PVA hydrogel.

Preparation of PDMA hydrogel. DMA (240 mg) and stock solutions of MBAA (43 µL, 10 mg/mL) and KPS (74 µL, 5 mg/mL) were dispersed in DI water (884 µL) under vortex to make a homogeneous suspension. The aqueous solution was then degassed by sparging under nitrogen under an ice bath for 20 min to remove oxygen and then placed in a 70 °C oven for 5 h to form a monolithic hydrogel.

Preparation of gelatin hydrogel. Typically, gelatin (20 wt %) was dissolved in DI water at 50 °C for 10 min to get a homogenous solution. The solution was subjected to a freeze/thawing treatment for 1 and 12 h respectively to form a gelatin hydrogel.

Preparation of PDMA coated hydrogel. PDMA coated gel was prepared using the same protocol as described in PHPMA coated hydrogel in the previous section except the monomer is DMA instead of HPMA. PDMA polymer was not precipitated during the polymerization process. Therefore, it formed a transparent hydrogel coating (see **Figure S4**) instead of porous polymer film. This hydrogel coating can be peeled off from the Agar/PAAm hydrogel substrate.

Film thickness measurement. Film thickness of PHPMA or PDAAM coatings were measured by a surface stylus profilometer (Veeco Dektak 8) with a stylus force of 3 mg and a travel speed of 60 µm/s. To form a patterned polymer coating on a blank agar/PAAm hydrogel substrate, a cylindrical PETE mould with a predesigned round shaped cylindrical hole (radius = 4 mm and thickness = 5 mm) was prepared using a laser-cutting machine (Figure 3a in the main text). As a template, the mould was applied onto the gel to cover the entire hydrogel surface treated with APS solution as previously described. Thereafter, HPMA or DAAM monomer solution was added into the hole for several minutes (typically 10 min), during which the round shaped polymer pattern was formed and bonded onto hydrogel surface. For the preparation of multilayer polymer pattered hydrogel samples, the procedure is identical to the abovementioned procedures except that we soaked the 1-layer coated hydrogel again in APS solution for 5 min and immersed the APS loaded gel in the HPMA solution for 10 min to grow the second layer. The third and fourth layers were grown from the second and third layers, respectively, using identical protocols.

PHPMA polymer pattern growth on hydrogel. To form a robustly bonded circuit and ‘UNSW’ shaped patterns on flat hydrogel surfaces, two masks were prepared using laser cutting (see **Figure S3**). The preparation is identical to previous described procedure except for the substrates are flat Agar/PAAm hydrogel.

Polymer film growth on porcine skin. Based on the experiments above, we further established a porcine model with removal of epidermis as a proof-of-concept to evaluate the further potential applications of this technique in clinical area, such as skin tissue replacement. A fresh porcine skin sample (pork tenderloin, 20 × 12 × 6 mm) with the reservation down to fat layer was cut using a razor blade to remove the epidermis layer (~ 0.5 mm). Then, similar to the hydrogel coating procedures, the porcine sample was immersed in APS solution for 5

min and submerged in the HPMA precursor solution for 10 min. The sample was washed with distilled water three times and pat-dried to remove excess surface water for subsequent SEM analysis.

Physical coating of PHPMA coated hydrogel with DDA. DDA (6 g) solid powder was heated up to 45 °C to form a clear solution. A PHPMA coated hydrogel sample was immersed into the solution for 30 s. After removed from the solution, the gel was washed with DCM three times after liquid DDA solidified at room temperature (~10 s). The collected gels were subjected to water loss testing.

Water loss testing. Hydrogel dehydration (cylindrical, diameter = 12 mm, thickness = 8 mm) was evaluated under ambient conditions (24 °C and 30 % relative humidity) for different time. The weights (W) of all samples were recorded at regular intervals for comparison. The water loss ratios were calculated by using the following equation:

$$W_L = (1-W/W_0) \times 100\ \% \qquad \text{(Eq.1)}$$

Where W_L, W and W_0 represent the water loss ratio, measured weight and original weight of the samples, respectively.

Fabrication of v-AuNWs@SEBS sensor: Firstly, the SEBS elastomer dissolved in toluene (80 mg/mL) was spin-coated on a clean silicon wafer at 1000 rpm for 30 s to prepare a SEBS film. After curing in the oven (60 ℃) for 30 min, the photoresist AZ1512 was spin-coated on the top of SEBS film at 3000 rpm for 45 s. A pattern was formed via a conventional photolithography process with a pre-designed photomask to expose the SEBS. v-AuNWs was grown on the exposed SEBS using a modified protocol of seed-mediated approach.[1] Briefly, 2 nm gold seed was synthesized by mixing 0.25 mL 25×10^{-3} M gold (III) chloride trihydrate and 0.147 mL 3.4×10^{-2} M sodium citrate in 20 mL H_2O in a conical flask. After one minute vigorous stirring, 600 µL ice-cold 0.1 M $NaBH_4$ solution was added in the solution, which was stirred for 5 min until the color turned from light yellow to red. The gold seed solution was stored at 4 °C prior to further use. To grow v-AuNWs, the exposed SEBS surface was treated with O_2 plasma for 5 min. The silicon wafer was then immersed in a 5×10^{-3} M (3-aminopropyl)triethoxysilane aqueous solution for 2 h to functionalize the SEBS surface with amino groups. After rinsing with DI water two times, the amino functionalized surface was immersed into citrate-stabilized gold seed solution for 2 h, followed by rinsing with DI water two times to remove excess seed particles. Lastly, the Au seed-modified substrates were

immersed in a growth solution containing 9.8 × 10^{-4} M 4-mercaptobenzoic acid, 1.2 × 10^{-2} M gold (III) chloride trihydrate, and 2.9 × 10^{-2} M *L*-ascorbic acid for 5 min, resulting in the formation of the v-AuNWs thin film. After growth of v-AuNWs, photoresist was removed by flushing the silicon wafer with ethanol followed by drying under nitrogen gas.

Fabrication of aligned cracks: To fabricate sharpness sensitive strain sensors, aligned cracks were generated on the outside pattern of the v-AuNWs@SEBS sensor. The cracks were generated by the method reported in the previous work.[2] Firstly, the v-AuNWs@SEBS sensor was transferred on a thick PDMS layer for sputtering and crack generation. A shadow mask was covered on top of v-AuNWs to expose the outside pattern. A layer of silver thin film was sputtered with speed of 0.3 nm s^{-1}. Localized channel cracks could be formed on v-AuNWs after applying a repeated strain of ~10% for 10 cycles using a uniaxial moving stage (THORLABS Model LTS150/M). Silver thin film was then dissolved by hydrogen peroxide/ammonium hydroxide (1:1) solution.

Encapsulation and hole punch of the free sensor: After the fabrication of cracks in v-AuNWs, the as-prepared v-AuNWs/SEBS sensor was encapsulated by another layer of SEBS (spin-coating at 1000 rpm for 30 s). The resulting sensor was finally transferred onto a water-soluble PVA tape by peeling it off from the PDMS. The uniformly distributed holes with diameter of 1 mm on the sensor was created manually by using a hole puncher (d = 1 mm).

Pressure and temperature sensing performance: A cylinder-shaped (d=10 mm) PDMS probe is attached to a force gauge (Mark-10 M7-2) equipped to a test stand (Mark-10, ESM 301 L), the hydrogel electronic skin (E-skin) was put on the test stage. Uniform pressure cycles were applied by a computer-based user interface, while the force and current readouts were measured by the force gauge and an electrochemical system (PARSTAT 4000 A, Princeton Applied Research) simultaneously. For temperature sensing, the sensor was fixed near a hot plate with adjustable temperature. The surface temperature was recorded by a portable infrared temperature detector.

Pressure sensing performance test using three probes with different sharpness: For the probe sharpness test, three cylinder-shaped PDMS probes with different contact area (R = 5， 2 and 0.5 mm) were attached to the force gauge (Mark-10 M7-2) equipped to a test stand (Mark-10, ESM 301 L). All other steps are the same as pressure sensing performance characterization.

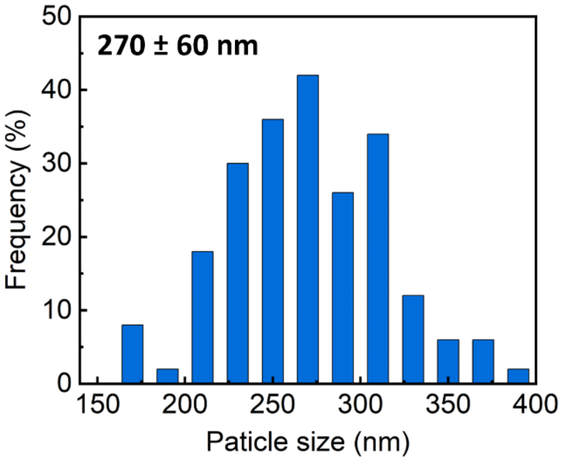

Figure S1. PHPMA particle size distribution on the surface of coated gel synthesized at 10 min polymerization time.

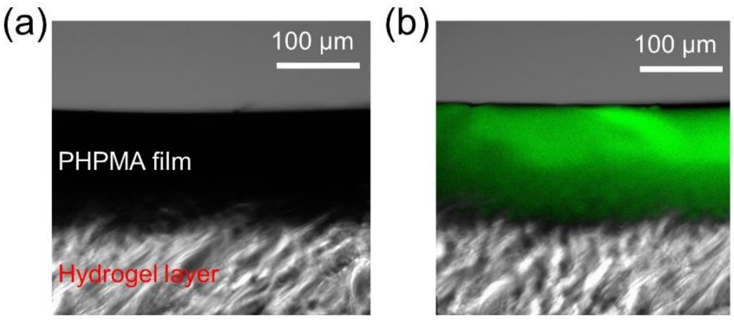

Figure S2. Fluorescence microscopy of PHPMA coated hydrogel with FA labelling. (a) Bright field image of the cross-section of the PHPMA coated hydrogel, showing the dark area as the PHPMA film. The film thickness is around 140 μm. (b) Merged photo of bright field and fluorescent images of PHPMA coated hydrogel with fluorescent (FA) labelling.

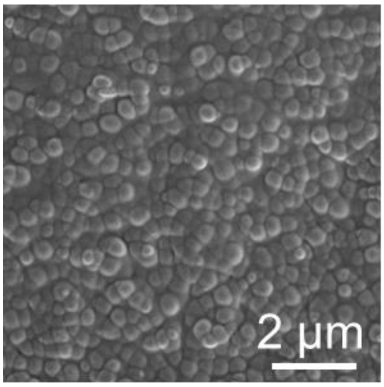

Figure S3. A SEM image for the cross-section of nanoparticle/hydrogel hybrid interface layer, showing that the polymer nanoparticles are progressively smaller but fused together.

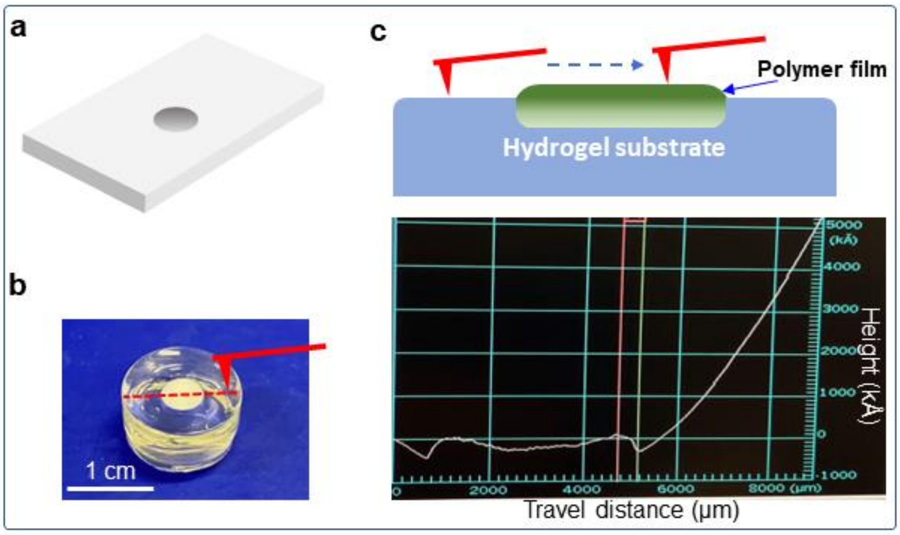

Figure S4. Thickness measurement of porous polymer film using a stylus surface profilometer. (a) A mask with a cylindrical hole (4 mm diameter and 5 mm thick) in the middle; (b) Digital photo for a hydrogel with a patterned PHPMA coating for surface profilometry; (c) Height profile of surface patterned hydrogel along the red line indicated in **Fig. S4b**.

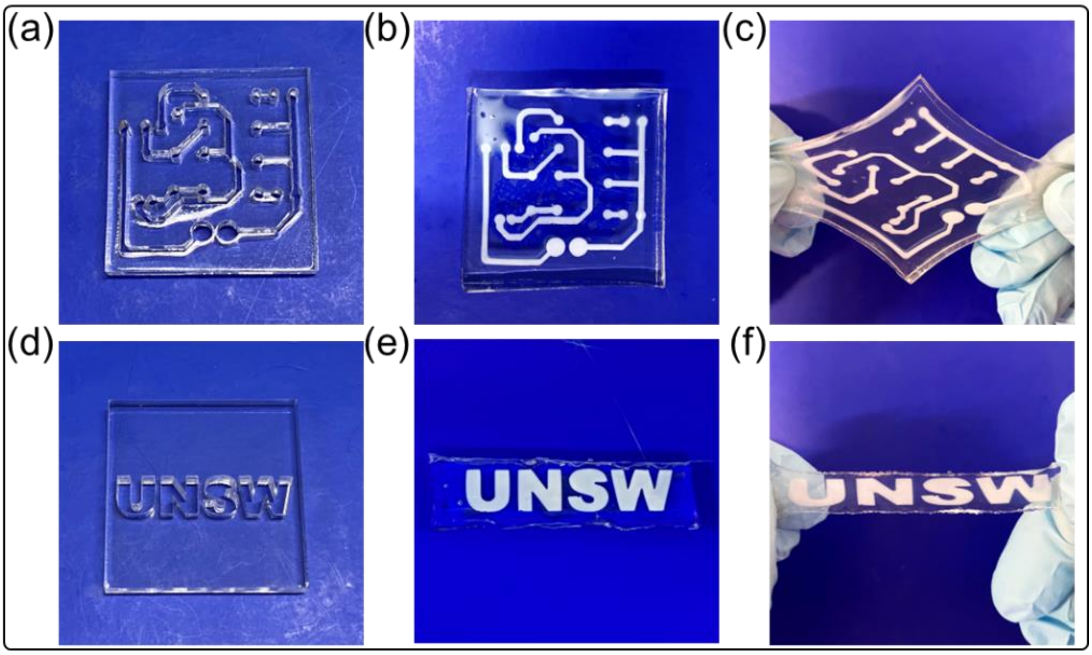

Figure S5. PHPMA polymer patterns produced on agar/PAAm hydrogels using masks. (a) A mask with circuit pattern; (b) PHPMA circuit pattern coated on a hydrogel substrate; (c) The hydrogel with a circuit pattern under deformation; (d) A mask with 'UNSW' pattern; (e) PHPMA pattern with the characters 'UNSW' on a strip of hydrogel substrate; (f) The hydrogel with a 'UNSW' pattern under deformation.

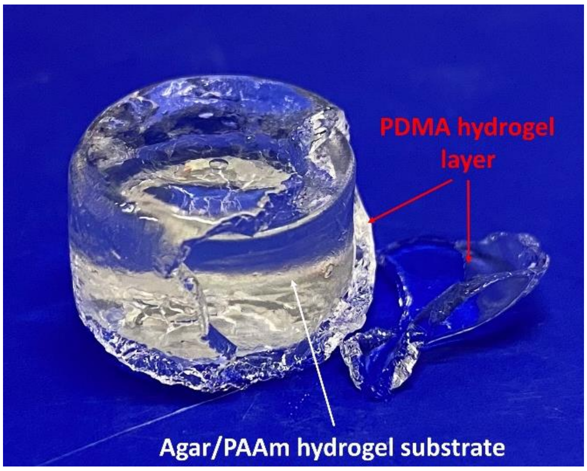

Figure S6. PDMA coated gel formed with same protocol and monomer concentration.

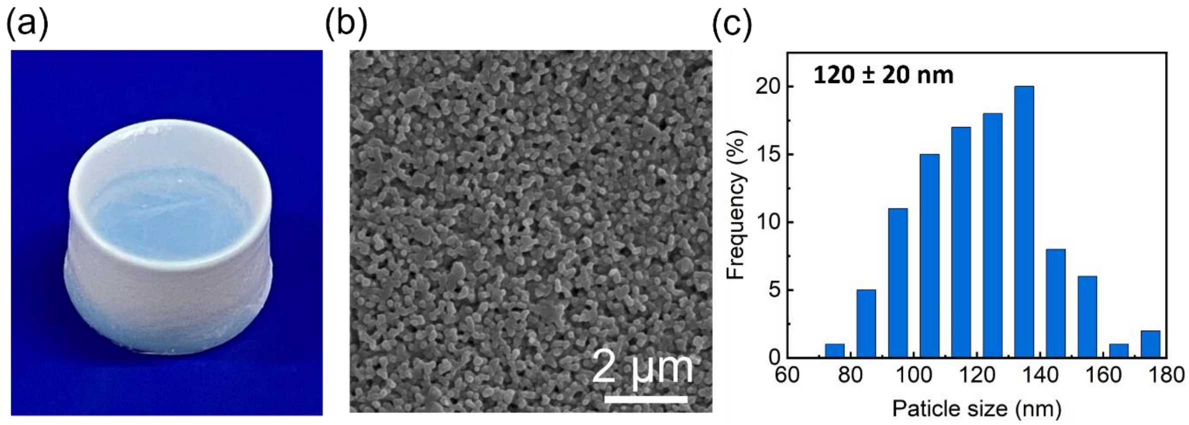

Figure S7. Microscopic characterization of PDAAM coated hydrogel. (a) Optical image and (b) SEM image for surface morphology of PDAAM coated hydrogel; (c) PDAAM particle size distribution on the surface of coated gel based on the SEM image.

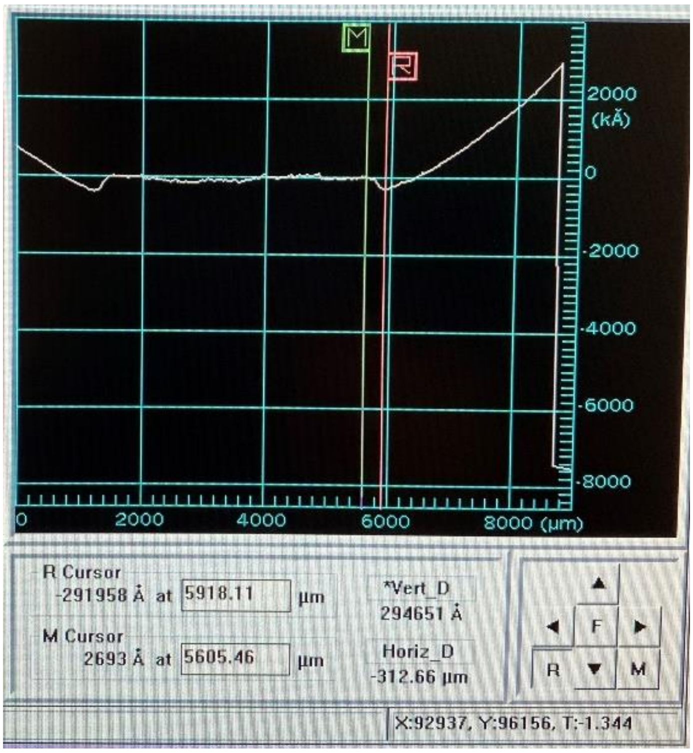

Figure S8. Height profile of PDAAM surface patterned hydrogel prepared by a mask with a cylindrical hole (4 mm diameter and 5 mm thick) in the middle measured by a stylus surface profilometer.

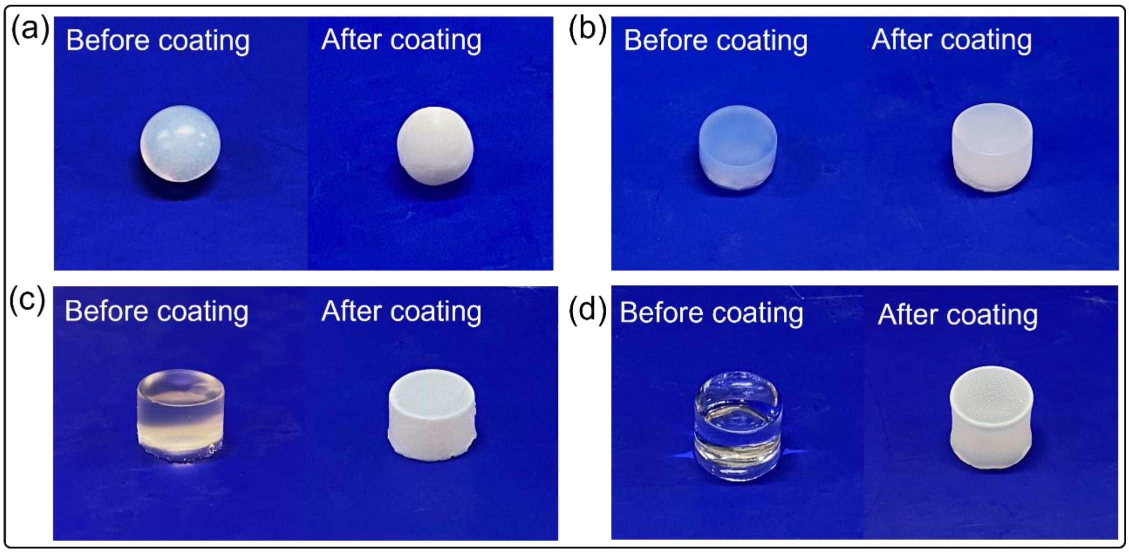

Figure S9. Optical images for PHPMA coated hydrogels before and after coating, (a) alginate; (b) PVA; (c) gelatin; and (d) PDMA.

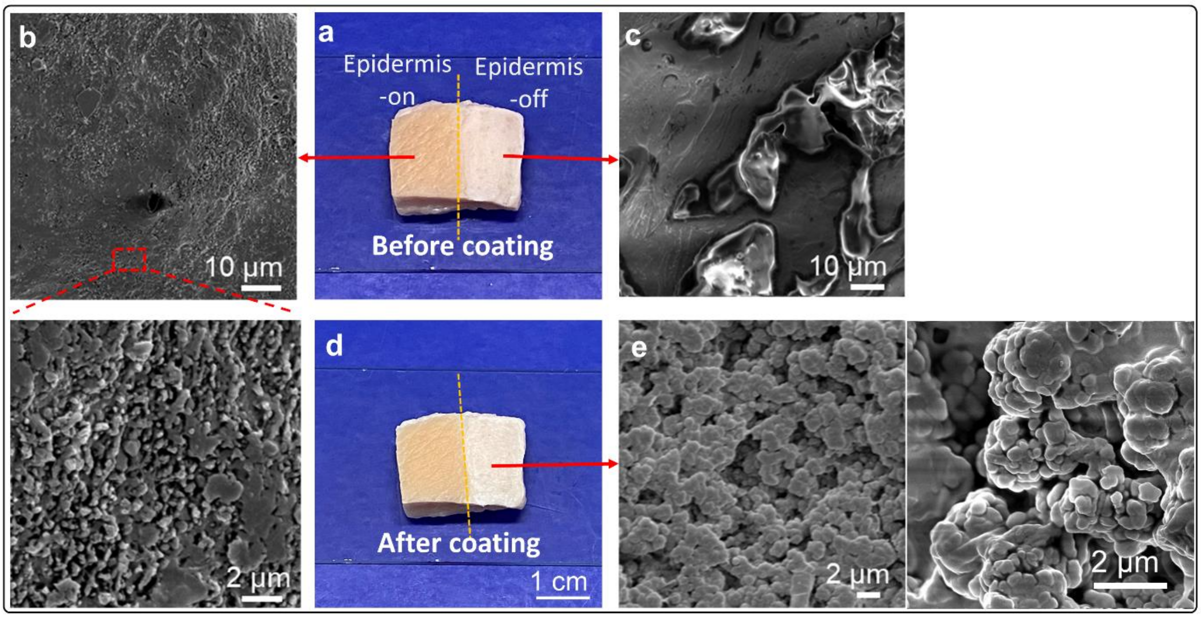

Figure S10. Growth of PHPMA film on porcine skin and SEM characterization on surface morphology before and after coating. a, d) Optical images for porcine skin before and after polymer coating. b) SEM images for surface morphology of epidermis-on skin. c, e) SEM images for surface morphology of epidermis-off skin before and after polymer coating. The nanoparticles have the size of 200-300 nm.

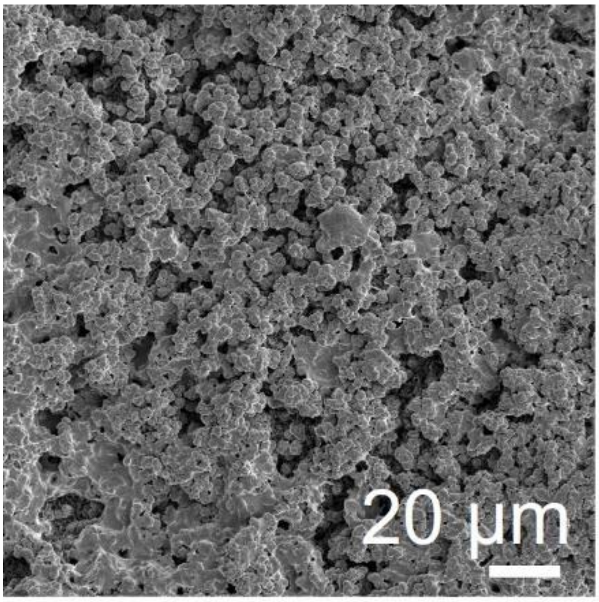

Figure S11. Surface morphology of PHPMA coated porcine skin with the epidermis removed after multiple bending, showing intact polymer film on the surface.

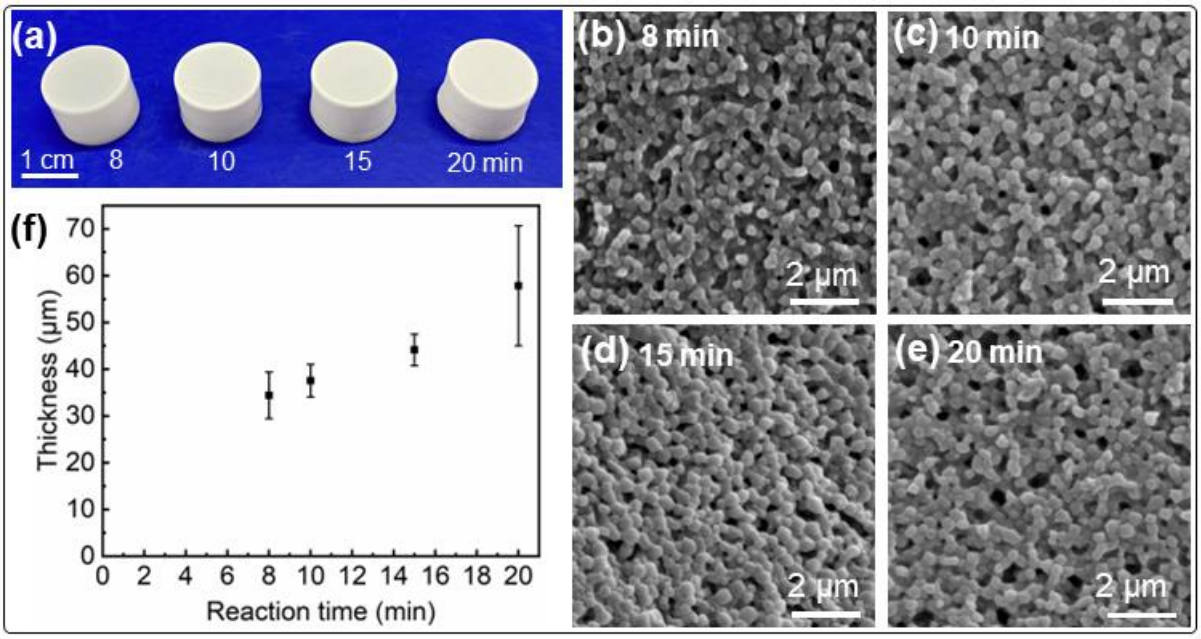

Figure S12. Microscopic and thickness measurement of PHPMA coated hydrogels prepared at different polymerization time. (a) Optical image of the gels; SEM images for the surface morphologies of the gels at (b) 8 min; (c) 10 min; (d) 15 min; (e) 20 min; (f) film thickness at different reaction time. The thickness values are averaged by measuring four different samples (error bars are included).

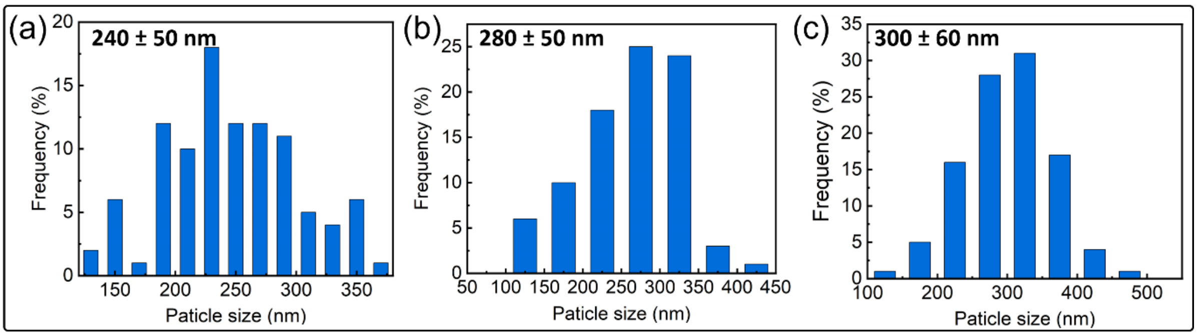

Figure S13. PHPMA particle size distribution on the surface of coated gel synthesized at different polymerization time (a) 8 min; (b) 15 min; (c) 20 min.

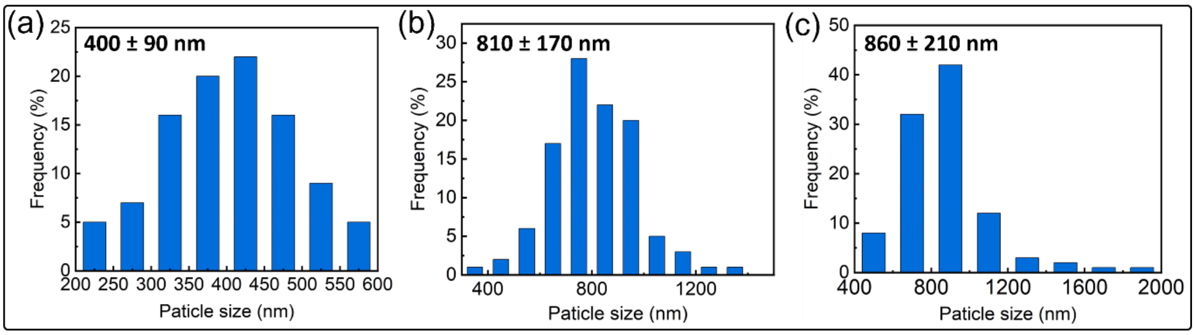

Figure S14. PHPMA particle size distribution on the surface of multi-layered hydrogels. (a) 2 layers; (b) 3 layers and (c) 4 layers.

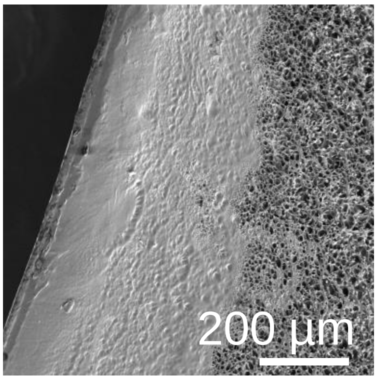

Figure S15. SEM image for cross-sectional morphology of PHPMA coated hydrogel with four layers of coating.

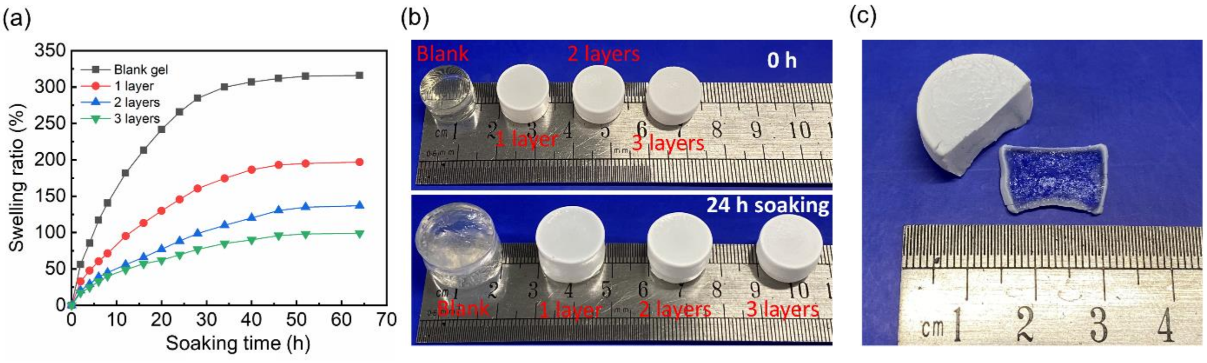

Figure S16. Swelling behaviour of the PHPMA coated hydrogels with different coating thickness in excess water. (a) Plot of swelling ratio vs soaking time in water for a blank hydrogel and three PHPMA coated hydrogels with different thickness (1 layer for 38 μm, 2 layers for 180 μm, and 3 layers for 250 μm). (b) Optical images for the blank and PHPMA coated hydrogels after soaking in excess water for 0 h and 64 h. (c) Optical image of cross-sectional PHPMA coated hydrogel with a 250 μm thickness (3 layers) after soaking in excess water for 64 h.

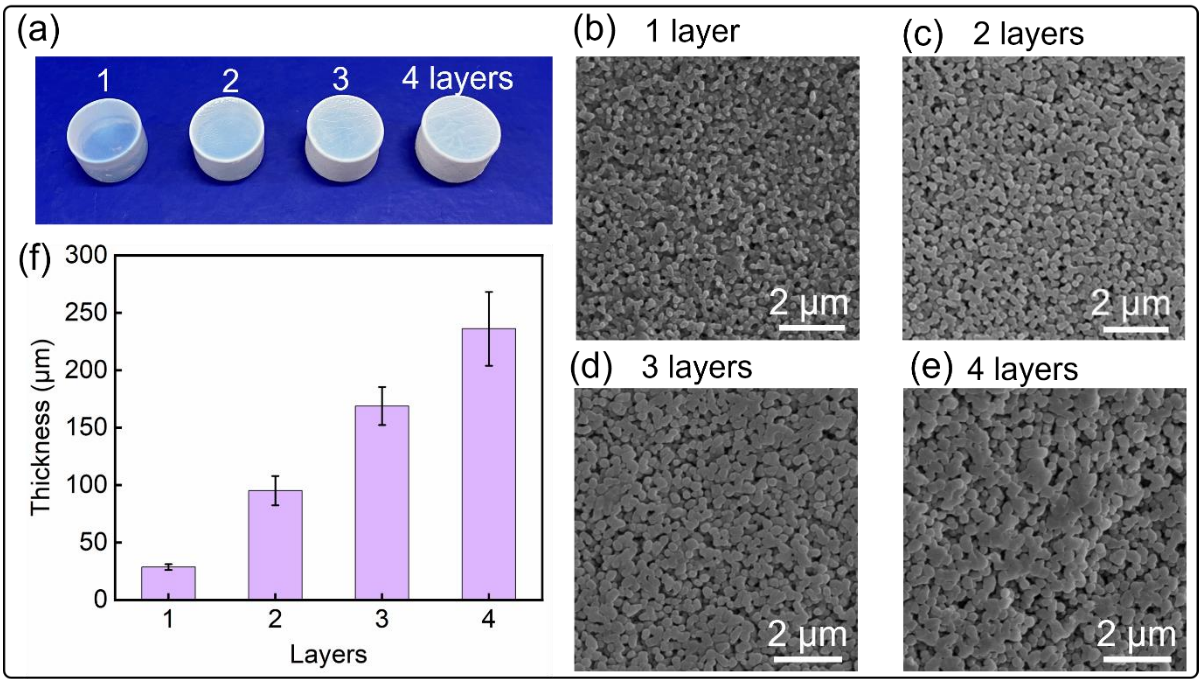

Figure S17. PDAAM coated hydrogels with different layers of coating. (a) optical image; SEM images for surface morphologies of the hydrogels with (b) 1 layer; (c) 2 layers; (d) 3 layers; and (e) 4 layers of PDAAM coatings; (f) thickness of different layers.

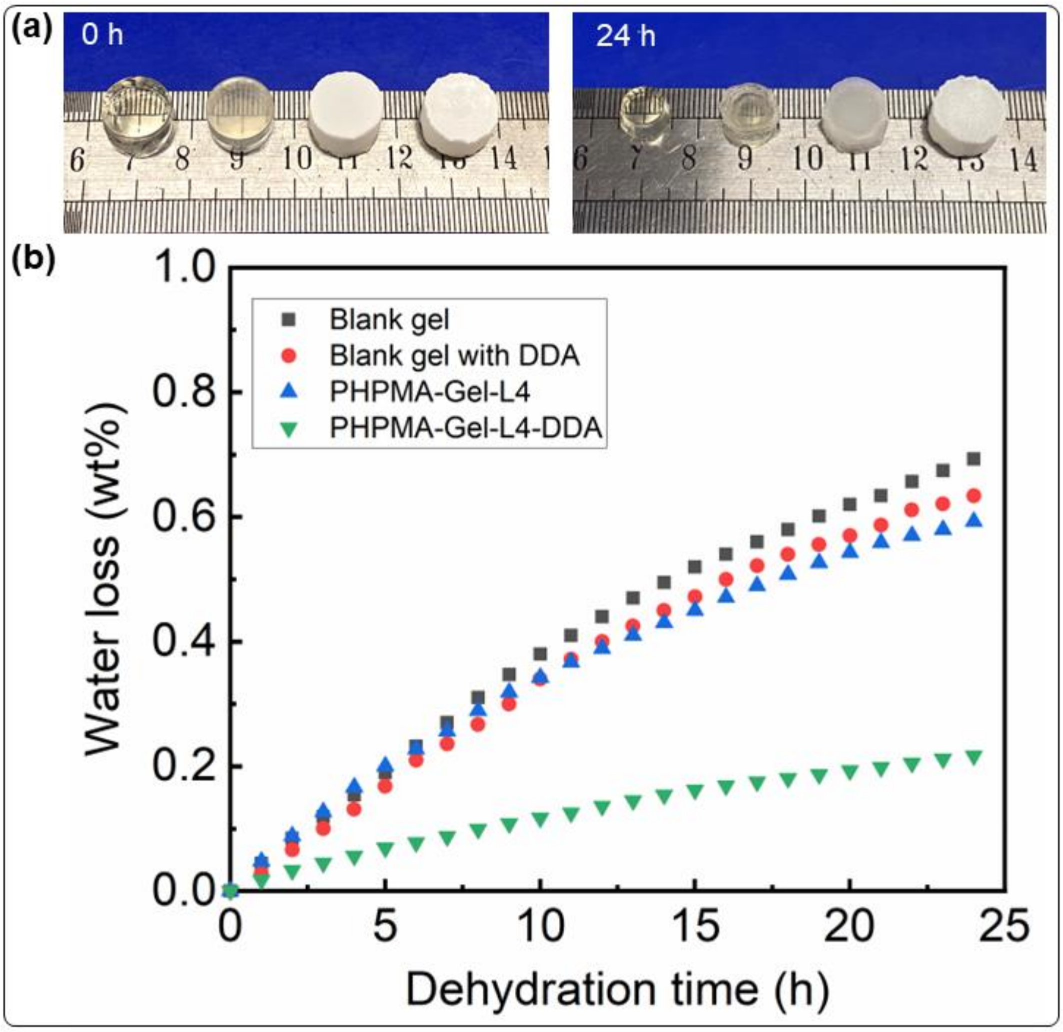

Figure S18. Water loss test of PHPMA coated Agar/PAAm hydrogels treated physically by dodecylamine (DDA). Optical images for the treated hydrogels after (a) 0 h and 24 h dehydration; (b) anti-dehydration performance. 'PHPMA-Gel-L4' is the short name for 'PHPMA coated hydrogel with a four-layer coating'. 'PHPMA-Gel-L4-DDA' means the sample of PHPMA coated hydrogel with a four-layer coating after 24 h curing with DDA.

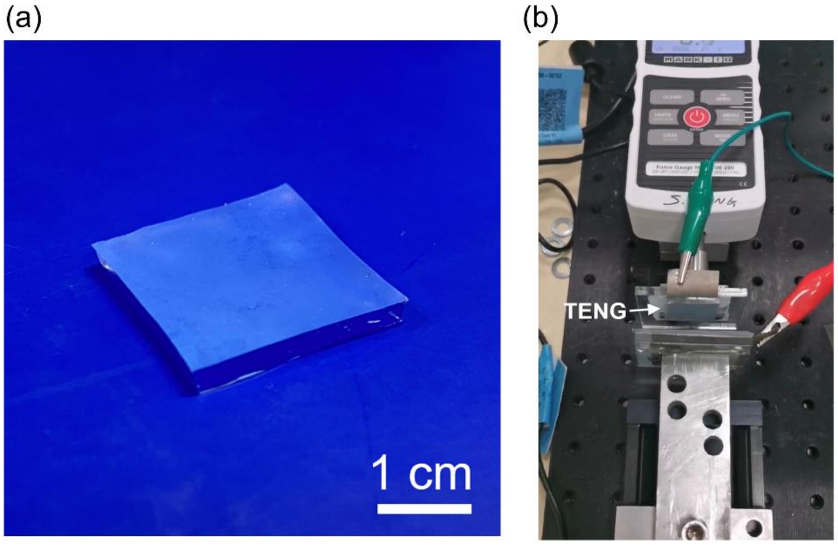

Figure S19. PDAAM coated hydrogel (with 8 M LiCl) as the TENG device for pressure sensing. a) Digital photo for the PDAAM coated hydrogel. b) TENG measurement in contact-separation mode.

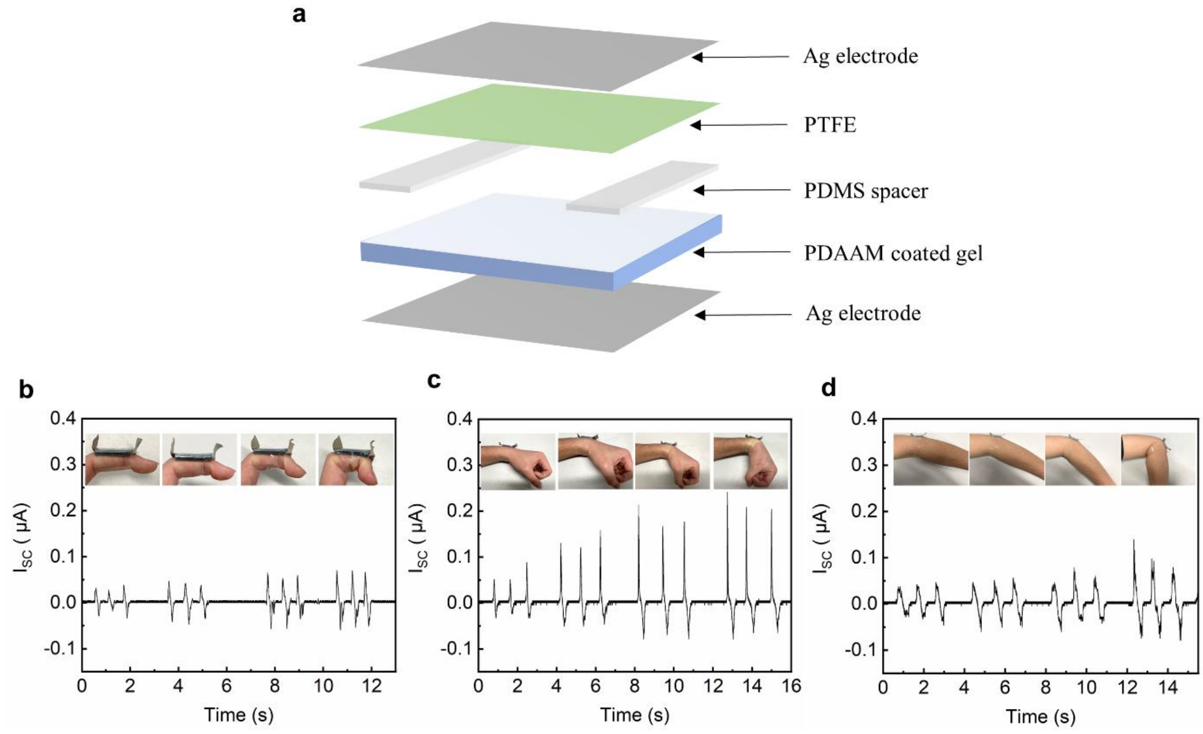

Figure S20. PDAAM/hydrogel-TENG device for body motion detection.

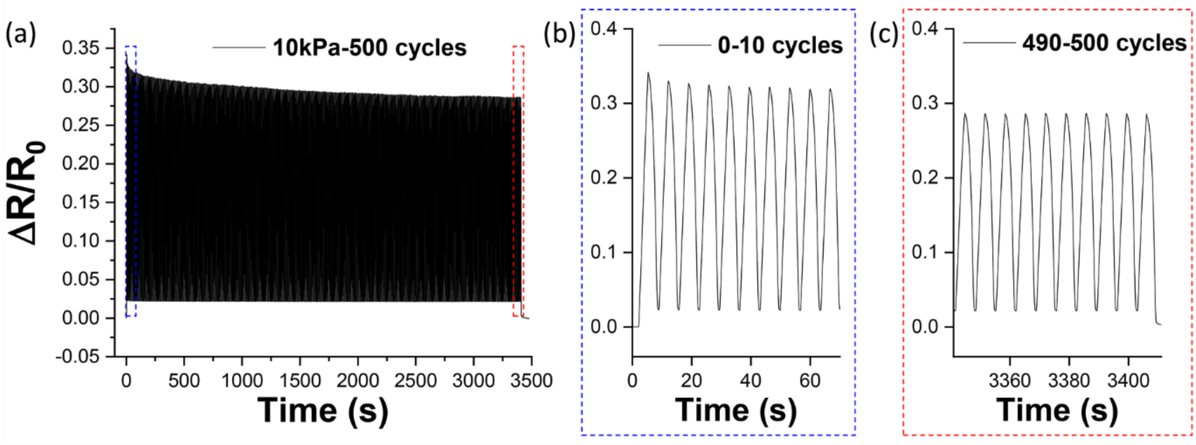

Figure S21. (a) Resistance changes of a v-AuNWs/SEBS pressure sensor during a durability test for 500 cycles with repeated 0-10kPa-0 pressure. (b), (c) are enlarged views of the first 10 (b) and the last 10 cycles (c).

1. Wang, Y.; Gong, S.; Wang, S. J.; Yang, X.; Ling, Y.; Yap, L. W.; Dong, D.; Simon, G. P.; Cheng, W., Standing Enokitake-Like Nanowire Films for Highly Stretchable Elastronics. *ACS Nano* **2018,** *12*, 9742-9749.
2. Gong, S.; Yap, L. W.; Zhu, B.; Zhai, Q.; Liu, Y.; Lyu, Q.; Wang, K.; Yang, M.; Ling, Y.; Lai, D. T. H.; Marzbanrad, F.; Cheng, W., Local Crack-Programmed Gold Nanowire Electronic Skin Tattoos for in-Plane Multisensor Integration. *Advanced Materials* **2019,** *31*, 1903789.
